# Levels of Potentially Toxic and Essential Elements in Water and Estimation of Human Health Risks in a River Located at the Interface of Brazilian Savanna and Amazon Biomes (Tocantins River)

**DOI:** 10.3390/toxics12070444

**Published:** 2024-06-21

**Authors:** Thiago Machado da Silva Acioly, Marcelo Francisco da Silva, Letícia Almeida Barbosa, José Iannacone, Diego Carvalho Viana

**Affiliations:** 1 Postgraduate in Animal Science (PPGCA/UEMA), Multi-User Laboratories in Postgraduate Research (LAMP), State University of Maranhão, São Luís 65081-400, Brazil; tmsacioly@gmail.com (T.M.d.S.A.); lealmeid.barbosa@gmail.com (L.A.B.); 2 Center for Exact, Natural and Technological Sciences (CCENT), State University of the Tocantina Region of Maranhão (UEMASUL), Imperatriz 65901-480, Brazil; silvamf@uemasul.edu.br; 3 Laboratorio de Ecología y Biodiversidad Animal (LEBA), Grupo de Investigacion de Sostenibilidad Ambiental (GISA), Facultad de Ciencias Naturales y Matemática, Universidad Nacional Federico Villarreal, Lima 15007, Peru; joseiannacone@gmail.com; 4 Center of Agrarian Sciences, Center for Advanced Morphophysiological Studies (NEMO), State University of the Tocantina Region of Maranhão (UEMASUL), Imperatriz 65900-000, Brazil

**Keywords:** biomonitoring, drinking water quality, environmental quality, pollutant, heavy metals

## Abstract

The Tocantins–Araguaia basin is one of South America’s largest river systems, across three Brazilian states (Maranhão, Tocantins, and Pará), within the Legal Amazon region. Despite draining extensive Cerrado savanna and rainforest ecosystems, it has suffered significant degradation, notably in the past 40 years. Human activities, including agricultural expansion, deforestation, and the introduction of non-native species, have worsened the environmental damage, which is alarming since many residents and villages along the middle Tocantins River rely on it for water supply, recreation, and fishing. This study assessed the concentration of potentially toxic and essential elements in water samples from four sampling sites distributed along the middle Tocantins River. The monitoring occurred throughout 2023, involving the measurement of parameters both on-site and in the laboratory. Water quality and its health implications were evaluated using the Weighted Arithmetic Water Quality Index (WAWQI), the Water Quality Index (WQI), and the health risk assessment index. The levels of aluminum, copper, iron, magnesium, and selenium exceeded legal standards. Seasonal fluctuations indicate a complex dynamic influenced by climatic or seasonal factors, with February showing the highest values. Site P1, located in urban areas, exhibited elevated mean concentrations for conductivity, total dissolved solids (TDS), and chlorophyll, indicating the need for continuous monitoring. The nitrogen concentrations at P1 raise concerns regarding drinking water quality, which is a concern for the region’s residents who use untreated river water. Despite seasonal variations in element concentrations, the overall WAWQI categorized all sections as “Excellent,” and the WQI rated as “Good.” Human health risk assessments detected no risks, but continuous monitoring and interventions are crucial for sustained water quality improvement.

## 1. Introduction

Contaminated water poses significant risks to human health, leading to waterborne diseases and impacting communities and healthcare systems. Access to clean and safe water directly correlates with improved global health indicators [1,2]. Despite efforts to expand access to clean drinking water, millions of people around the world still suffer from preventable illnesses due to contamination, emphasizing water’s critical role in sustaining human health [3]. Consequently, the declining water quality not only affects the environment but also imposes substantial economic and public health burdens [4,5].

Anthropogenic impacts on aquatic ecosystems stem from diverse sources, including industrial, urban, agricultural, and mining pollution [6,7]. Human interventions, such as dam construction, salinization, deforestation-driven sedimentation, riparian vegetation clearance, and intensive fishery exploitation, further exacerbate this imbalance [8]. Consequently, contaminants from these activities disrupt the ecosystem equilibrium when introduced into aquatic environments. Notably, approximately 80% of infections in low-income and developing countries are directly attributable to contaminated drinking water and unhygienic settings [9]. Potentially toxic and essential elements, sourced from domestic, agricultural, and industrial waste, pose hazards to humans, marine life, and the environment [10]. Heavy metal pollution, particularly prevalent in developing countries, is a global concern affecting rivers worldwide [11,12,13]. 

Nitrogenous compounds such as total ammoniacal nitrogen (NH_3_^−^NH_4_^+^) from agricultural and industrial effluents significantly contribute to groundwater pollution [14,15]. Understanding these processes is crucial for effective water resource management and the implementation of preventive measures to mitigate pollution impacts. Nitrogen exists in water in various forms, and careful monitoring of its levels is crucial due to the potential for excess. Elevated ammonium levels, often resulting from excessive wastewater-based chemical usage, have been associated with eutrophication [16]. This condition increases water purification costs, stimulates algal growth, poses health risks to humans and livestock, diminishes the recreational value of water bodies, causes objectionable changes in aquatic environments, and leads to oxygen depletion [17,18,19].

After persistent pollutants are released into the environment, they can accumulate in various environmental compartments, such as water, sediment, and biota, in different chemical forms, posing a threat to human health through multiple absorption pathways [20,21]. These pathways for human exposure to contaminants include the soil–food chain, skin contact, inhalation, and oral intake. Excessive dietary accumulation of potentially toxic elements in the human body can lead to severe systemic health problems [22,23], emphasizing the importance of addressing this type of contamination and its associated health risks.

Water quality monitoring typically generates a large and intricate database that includes biological, physical, and chemical variables, making it challenging to manage [24]. To address this complexity, the suitability of water sources for human consumption has been assessed using the Water Quality Index (WQI), which is considered an effective form of characterizing water quality. Water quality assessment employs various methods, with the Weighted Arithmetic Water Quality Index (WAWQI) and the Water Quality Index (WQI) standing out as prominent examples of multiple objective decision-making methods [25,26]. The WQI offers a simplified approach but may not fully capture all nuances of water quality. In contrast, the WAWQI enables a more detailed and customized assessment by assigning weights to parameters based on their relative importance to water quality and human health [27,28]. This approach can provide a more accurate and specific assessment of water quality by considering the relative importance of each parameter.

Among the largest river systems in South America, the Tocantins–Araguaia basin stands out due to its extensive drainage area of 767.000 km^2^, traversing three Brazilian states (Maranhão, Tocantins, and Pará) and falling within the Legal Amazon region [29,30]. Additionally, this river’s ichthyofauna is highly diverse, comprising approximately 300 species, 126 genera, and 34 families, primarily Characiformes (order), Siluriformes (order), and Cichlids (family) [31]. Although the Araguaia–Tocantins basin is not directly connected to the Amazon River, it shares an estuarine region with it [32]. This basin drains a vast area of the Cerrado savanna and rainforest ecosystems, known for their large and biodiverse floodplains [33]. However, this basin has experienced significant degradation, particularly over the last 40 years, primarily due to the expansion of dams, croplands, irrigation, mining, and aquaculture [33].

The middle Tocantins River region has seen a surge in various human activities, from agricultural expansion to deforestation, along with the introduction of non-native species, exacerbating environmental degradation. Around 32.244 individuals (12% of the population) lack access to clean water at home, while a staggering 181.533 lack a sewage system (69.83%) [34]. Due to this shortfall, many residents dispose of domestic waste directly into the river. Despite using the same untreated water for essential tasks such as bathing, laundry, and even consumption, the absence of proper sanitation facilities poses significant health risks. In many rural or low-income areas, people place pumps on the riverbanks to draw untreated water, exposing individuals to chemical and biological contaminants. This degrades water quality and increases disease risks for those dependent on the river, highlighting the necessity of appropriate mitigation measures to protect public health. 

Starting in the 1970s and 1980s, population growth and waste accumulation began to degrade the quality of streams that traverse the city and feed into the Tocantins River [35]. For example, the Bacuri stream lacks proper banks, causing houses to be built in the water and leading to unsanitary conditions as household waste, especially from bathrooms and kitchens, is dumped directly into the stream, turning it into an open sewer [36]. This stream has shown concentrations of metals such as Cu, Pb, Fe, and Cr exceeding national standards, indicating that these elements are being carried to the Tocantins River as suspended particulate matter [37]. It is worth noting that fish sourced from the Tocantins River, near Imperatriz city, serve as a primary protein source for riverside dwellers, albeit with observed mercury concentrations averaging 0.2775 µg/g for dogfish and 0.1360 µg/g for mapará [38]. Many riverside residents in the region and neighboring villages depend on the waters of the Tocantins River for direct household water supply, recreational purposes (bathing), and artisanal fishing for family consumption and/or commercialization.

The information gaps this study seeks to fill revolve around the lack of comprehensive studies and environmental monitoring in the middle Tocantins River. Essentially, this study assessed the concentration of potentially toxic and essential elements in water samples from this region. Additionally, it investigated various physicochemical parameters related to water quality (air and river water temperature, chlorophyll, total dissolved solids, pH, turbidity, conductivity, oxidation–reduction potential, luminescent dissolved oxygen, and salinity) and the presence of nitrogenous compounds (NH_4_^+^, NO_2_, and NO_3_^−^). The quality of water and its impacts on human health were also evaluated using the Weighted Arithmetic Water Quality Index (WAWQI), the Water Quality Index (WQI), and the health risk assessment index. The environmental fragility of the middle Tocantins River underscores the urgency for increased attention to environmental monitoring in this vital aquatic ecosystem.

## 2. Materials and Methods

### 2.1. Study Area and Sampling

**Figure 1:**
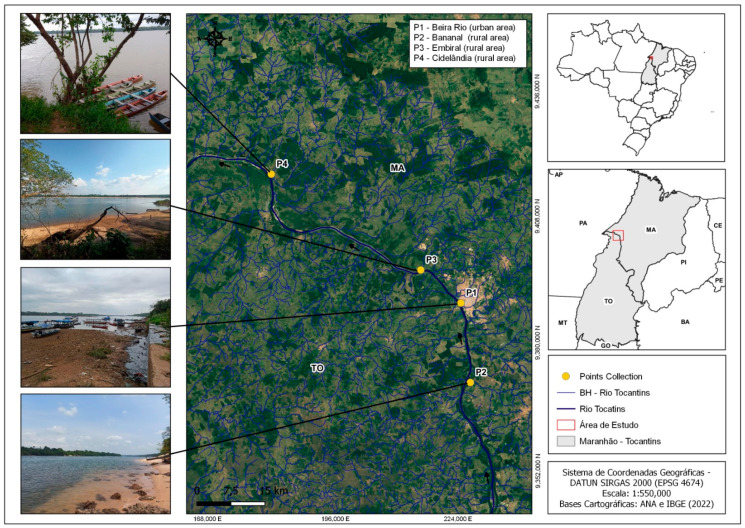
Location, main characteristics, and geographic coordinates of the sampling sites in the middle Tocantins River, Maranhão, Brazil. P1: Beira Rio (urban area), P2: Bananal (rural area), P3: Embiral (rural area), P4: Cidelândia (rural area).

This research was conducted in the middle Tocantins River, within the Imperatriz area of influence (Maranhão, Brazil) (Figure 1). This region holds strategic importance both regionally and nationally as a key transportation hub and gateway to the Amazon. Its thriving agribusiness and industrial sectors drive economic growth, providing employment and fostering development. The municipality serves as the headquarters of the southwestern metropolitan region of the state (5°31′32″ S; 47°26′35″ W) and stretches along the right bank (South-North) of the Tocantins River. The precipitation and temperature data in the region throughout the year are depicted in Figure S1. This allows for the identification of the rainiest/coldest period (January to June) and the hot/dry season (July to December) [39].

The level of inorganic contamination was monitored from January to December 2023 by measuring parameters both in situ and in the laboratory and collecting water samples from different sections of the middle Tocantins River. Four sampling sites were established, each with GPS georeferencing (Figure 1). Water samples were collected monthly, with three repetitions in each section, totaling 121,000 mL polyethylene containers that had been previously prepared and washed with milli-Q water. The samples were collected at a depth of 1.5–2 m, with a distance from the riverbanks of 5–10 m and 100 m between repetitions. The river had an average depth of 30 m, an average width of 500 m, and a flow volume of 13,600 m^3^/s. After being collected with the assistance of the Center for Advanced Morphophysiological Studies (NEMO), the containers were labeled and refrigerated at 4 °C until they reached the laboratory.

The first sampling site (P1) was located in front of “Beira Rio”, an urbanized and recreational area, and was susceptible to contamination due to factors such as heavy vessel traffic, river beaches, solid waste disposal, and urban sewage input. Conversely, the second sampling site (P2), known as “Ribeirãozinho”, is situated farther from the city (30 km) and was expected to exhibit a lower contaminant potential. The third site (P3) is situated upstream of the paper mill manufacturing plant, 20 km from the city in another direction, which could be a possible contamination source. Lastly, the fourth sampling site (P4), “Praia da Viração”, located approximately 100 km downstream from the city, is situated in the Cidelândia village and is affected by industrial activities, particularly from the paper and cellulose industry.

The region grapples with challenges such as deforestation driven by agricultural expansion, monoculture cultivation (e.g., Eucalyptus), and sanitation issues due to urbanization. These activities collectively degrade water quality, impacting ecological health and community well-being. Deforestation leads to increased soil erosion and sedimentation, resulting in water turbidity and pollutant introduction. Urbanization causes runoff from impermeable surfaces, carrying pollutants such as oils, heavy metals, and nutrients into water bodies. The Tocantins–Araguaia basin is currently the most targeted area for expanding agricultural activities, as stated in Presidential Decree 8447 of 2015, which created the MATOPIBA Federal Plan for the Development of the Brazilian Cerrado. Data from 2019 indicate that pastures and monocultures covered more than 42% of the basin [33]. Agricultural expansion has dramatically increased the use of pesticides [40,41], which invariably end up in aquatic ecosystems. Broad changes in land use have resulted in the elimination of riparian forests and modifications to hydrological dynamics [42,43,44].

These activities result in numerous discharges being emitted into the atmosphere and the aquatic system, causing harm to the health of the Tocantins River (both fauna and flora) and impacting the quality of life of the population dependent on the river. Threats are escalating as public policies continue to prioritize maximizing economic growth at the cost of environmental sustainability [33]. Fishing is a cultural activity that has been generating income for generations in the region [45]. It serves as a vital protein source for the population of Maranhão, which is the 5th state with the highest fish consumption in Brazil and also supplies the demands of the states of Pará, Tocantins, and Piauí [46,47].

### 2.2. Water Physicochemical Parameters and Presence of Nitrogenous Compounds

Physicochemical determinations were conducted monthly throughout 2023, in triplicate, covering the following parameters: air and river water temperature (°C), chlorophyll (µg/L), total dissolved solids (TDS) (g/L), pH, turbidity (NTU), conductivity (µS/cm), oxidation–reduction potential (ORP) (MV), luminescent dissolved oxygen (LDO) (mg/L), and salinity (ppt). These measurements were obtained in situ using the Hydrolab multiparameter probe (model SX751—SANXIN), totaling 12 readings for each parameter (4 sampling sites, with 3 repetitions each). 

Sampling occurred monthly, over 12 occasions throughout the year, to collect this comprehensive dataset. The water samples were filtered to detect the presence of NH_4_^+^, NO_2_, and NO_3_^−^. NH_4_^+^ was quantified using Nessler colorimetric analysis, with spectrophotometer readings at 450 nm. For NO_2_, the Alphanaphthylamine colorimetric method was employed, with spectrophotometer readings at 520 nm. NO_3_^−^ was determined by the brucine colorimetric method, with readings at 415 nm. 

### 2.3. Determination of Potentially Toxic and Essential Elements in Water

The concentrations of potentially toxic elements (aluminum—Al, antimony—Sb, arsenic—As, barium—Ba, cadmium—Cd, chromium—Cr, lead—Pb, lithium—Li, nickel—Ni, strontium—Sr, titanium—Ti, and silver—Ag) and essential elements (boron—B, selenium—Se, silicon—Si, phosphorus—P, copper—Cu, iron—Fe, calcium—Ca, cerium—Ce, potassium—K, magnesium—Mg, manganese—Mn, molybdenum—Mo, sodium—Na, vanadium—V, cobalt—Co, tin—Sn, and zinc—Zn) were determined using inductively coupled plasma emission spectrometry (ICP-EAS) (SHIMADZU, ICPE-9000, Kyoto, Japan). The analyses were conducted using 50 mL samples in Falcons, pre-filtered on qualitative film paper, following the analytical methodology of the U.S. Environmental Protection Agency (US EPA Method 3015A). Calibration standards and blanks were treated in the same manner. Mercury (Hg) analysis for the samples was conducted following EPA Method 6020A.

### 2.4. Comparison of Data with National and International Standards

The results from physicochemical parameters, nitrogen compounds, and potentially toxic and essential elements in water were compared with the National Environmental Quality Standards [48], which serve as a benchmark (Table 1). These standards establish specific limits for each substance within every classification, providing a comprehensive framework for water quality assessment and regulation. Additionally, comparisons were made with international standards, such as the World Health Organization [49,50] and the Environmental Protection Agency [51].

### 2.5. Weighted Arithmetic Water Quality Index (WAWQI) and Water Quality Index (WQI)

The WAWQI is an index that comprehensively assesses water quality by combining various characteristics. This approach uses a weighted average, assigning weights to each parameter based on its relative importance. Studies [51,52] recommend processing the data using Equation (1):

(1) $$\mathrm{WAWQI}=\sum_{i=0}^{n}\mathrm{QiWi}$$

The sub-quality index for each variable is denoted as Qi, with the specified variable’s weight unit represented by Wi. There were 13 physicochemical characteristics (n = 13): water temperature, pH, TDS, Al, Ba, Cd, Cr, Cu, Fe, Pb, Mn, Ni, and Zn. They are expressed in mg/L, except for water temperature and pH. To calculate the quality of each parameter (qi), Equation (2) is used:

(2) (qi)=(Ci/Si) × 100

where Ci represents the concentration of the parameter in the water sample, and Si is the established quality standard for that parameter. Additionally, the unit weight (Wi) for each parameter needs to be calculated using Equation (3):

(3) Wi=wi/∑wi

Wi represents the unit weight of the pollutant variable; n is the total number of pollutant variables; and wi is the weight of each parameter (temp. = 0.00007; pH = 0.00002; TDS = 0.00001; Al = 0.00623; Ba = 0.01245; Cd = 0.62253; Cr = 0.06225; Cu = 0.15563; Fe = 0.00208; Pb = 0.08893; Mn = 0.01245; Ni = 0.02490; Zn = 0.01245) [52]. Finally, one should multiply the unit weight (Wi) of each parameter by the parameter’s quality (qi) and sum the results to obtain the WAWQI. Based on these data, water quality is classified on a scale from excellent to unsuitable for consumption, with numerical values calculated using Equation (1). Values falling within the range of 0 to 25 are classified as excellent, while those between 26 and 50 are considered good. Bad quality is indicated by values ranging from 51 to 75, and very bad quality by values between 76 and 100. Any value exceeding 100 is deemed unsuitable for consumption.

The Water Quality Index (WQI) is an effective method for assessing water quality, determined by the calculated IQA value. This value can range from very poor (WQI < 25) to excellent (91 < WQI ≤ 100) [53]. Each parameter contributing to the WQI is assigned a specific weight based on its relative impact on water quality [54]. The parameters considered include pH, water temperature difference, LDO, turbidity, and NO3-. Equation (4) is used to determine the WQI [54].

(4) $$\mathrm{WQI}=\sum_{i=1}^{n}{\mathrm{qi}}^{\mathrm{wi}}$$

### 2.6. Quantitative Health Risk Assessment: Average Daily Intake (ADI), Target Hazard Quotient (THQ), and Hazardous Index (HI)

For metals and metalloid contamination in water, food, and soils, ingestion and dermal contact play the most important roles among the potential exposure pathways [55,56]. Furthermore, risk assessment entails evaluating adverse health effects on humans exposed to substances over a specified period [57]. In this investigation, health risk was assessed by measuring contamination levels in the water of the middle Tocantins River, Brazil. Both adults and children were considered in this analysis (Table 2). 

To calculate the exposure dose through ingestion of water (EXPing) (mg/kg/day), Equation (5) is utilized, while for exposure dose through dermal absorption (EXP-der) (mg/kg/day), Equation (6) is applied [60]. In this case, Cw represents the concentration of the chemical in water (mg/L), Kp is the permeability constant (1 cm/h), and the other terms are provided in Table 1.

(5) $$\mathrm{EXPing}=\frac{\mathrm{Cw}\;\times \;\mathrm{IR}\;\times \;\mathrm{EF}\;\times \mathrm{ED}}{\mathrm{Bw}\;\times \;\mathrm{AT}}$$

(6) $$\mathrm{EXPder}=\frac{\mathrm{Cw}\;\times \;\mathrm{SA}\;\times \;\mathrm{Kp}\;\times \;\mathrm{EF}\;\times \;\mathrm{ED}\;\times \;\mathrm{ET}\;}{\mathrm{Bw}\;\times \;\mathrm{AT}}$$

Equation (6) is utilized to determine the THQ index. EFr represents total exposure frequency, ED is exposure duration, WiR stands for rate of water ingestion, C denotes the average concentration of trace elements in water, RfD indicates oral reference dose according to the USEPA, BW represents mean body weight, and AT signifies mean exposure time. The oral reference doses (RfD) for the trace elements are as follows: 1 mg/kg/day for aluminum (Al), 0.40 mg/kg/day for copper (Cu), 0.8 mg/kg/day for iron (Fe), 0.14 mg/kg/day for magnesium (Mg), and 0.005 mg/kg/day for selenium (Se) [10,61,62].

(7) $$\mathrm{THQ}=\left(\frac{\mathrm{EFr}\;\times \;\mathrm{ED}\;\times \;\mathrm{WiR}\;\times \;C}{\mathrm{RfD}\;\times \;\mathrm{BW}\;\times \;\mathrm{AT}}\right)\times 10^{-3}$$

The target hazard quotient (THQ) assesses the non-carcinogenic risk level from pollutant exposure. THQ values below 1 indicate insignificant health threats, while THQ values ≥1 indicate potential health risks requiring corrective action [63]. The Hazardous Index (HI) is the cumulative sum of the Target Hazard Quotient (THQ) values for all trace elements present in an individual’s exposure. It is calculated using Equation (8). An HI value of ≥1 indicates a potential non-carcinogenic risk to human health [64].

(8) $$\mathrm{HI}=\Sigma \;\mathrm{THQ}=\mathrm{THQ}\left(\mathrm{iAl}\right)+\mathrm{THQ}\left(\mathrm{iCu}\right)+\mathrm{THQ}\left(\mathrm{iFe}\right)+\mathrm{THQ}\left(\mathrm{iMg}\right)+\mathrm{THQ}\left(\mathrm{iSe}\right)$$

### 2.7. Statistical Analysis

To evaluate significant differences in the average total concentration of toxic and essential elements across sampling stations or within the same station during various samplings, an Analysis of Variance (ANOVA) was employed. Before the analysis, normality was assessed using the Kolmogorov–Smirnov test, and variance homogeneity was assessed using Levene’s test. Means were compared using the Tukey test (<0.05). Data analysis was conducted using SPSS version 22. Additionally, principal component analysis (PCA) was utilized to investigate and interpret water quality data results, facilitating the grouping of information and visualization of hidden structures and relationships. PCA was performed using software PAST 4.03 (latest version 2020).

PCA was used to identify patterns or relationships among the concentrations of toxic and essential elements, physicochemical parameters, and the presence of nitrogenous compounds relevant to water quality. The data remained untransformed, and the results are expressed as correlations. Selection and interpretation criteria involved analyzing principal components and their respective weights or loadings, emphasizing those explaining the majority of data variability. The technique was employed to reduce data dimensionality and facilitate the interpretation of inter-variable relationships, enabling a comprehensive analysis of factors influencing water quality in the Tocantins River.

## 3. Results and Discussion

### 3.1. Water Physicochemical Parameters and Presence of Nitrogen Compounds

During the 2023 monitoring period, the observed average pH values were 7.23 (P1), 7.18 (P2), 7.41 (P3), and 7.00 (P4) (Table 3). The average pH values for each section conform to the National and International Environmental Quality Standards guidelines [48,49,50]. It is worth mentioning that water availability influenced this factor, with higher values being found during the dry season, explaining the wide range for some variables studied. Thus, during this period, there were months when maximum values exceeded legal standards. Furthermore, the spatial data collected throughout 2023 are available in the Supplementary Materials (Tables S1–S12).

The urban area (P1) showed elevated and statistically significant levels of conductivity (51.67 µS/cm), total dissolved solids (TDS) (0.0326 g/L), and chlorophyll (1.47 µg/L) (Table 3). However, these readings remained within CONAMA’s recommended limits. High conductivity values might lead to unpleasant taste and digestive issues [65]. Although turbidity did not exhibit statistically significant differences among the data, the values slightly surpassed the recommended levels. To provide a meaningful comparison, it is useful to look at turbidity levels in other, cleaner rivers. Typically, cleaner rivers have turbidity levels well below those observed, often less than 5 NTU, indicating clearer water with lower concentrations of suspended particles [49]. Elevated turbidity levels can indicate higher concentrations of bacteria, microalgae, nutrients, pesticides, or metals [66,67]. 

The pH, conductivity, salinity, and nitrite concentration are among the parameters that can serve as effective water quality indicators based on statistical analyses [68]. This type of analysis is essential for environmental biomonitoring studies [69,70], given the sensitivity of ichthyofauna to environmental variations and physical–chemical contamination involving dissolved organic and inorganic compounds. 

Site P1 exhibited elevated mean concentrations of NH_4_^+^ (3.96 mg/L) and NO_2_ (0.07 mg/L) (Table 3). The region has an open sewage system that directly disposes of untreated wastewater from the neighborhood into the middle Tocantins River (Figure 1), with an average daily flow of approximately 411.6 L per second (calculated based on city indices and the average flow rate of the collection area). Additionally, domestic wastewater serves as a significant source of inorganic nutrients, including nitrogen (NH_3_^−^N) and phosphorus (PO_4_^−^P) [71,72]. The NH_4_^+^ and NO_2_ levels exceed the established limits for drinking water, while NO_3_^−^ content falls below thresholds [49]. Therefore, wastewater must be treated to reduce contaminants, pollutants, and undesirable components before being discharged into freshwater or water sources [73]. Additionally, physicochemical parameters and nitrogenous compound data collected throughout the region are provided in Table S1.

### 3.2. Temporal Distribution and Annual Average Concentration of Potentially Toxic and Essential Elements in Water from the Middle Tocantins River

**Figure 2:**
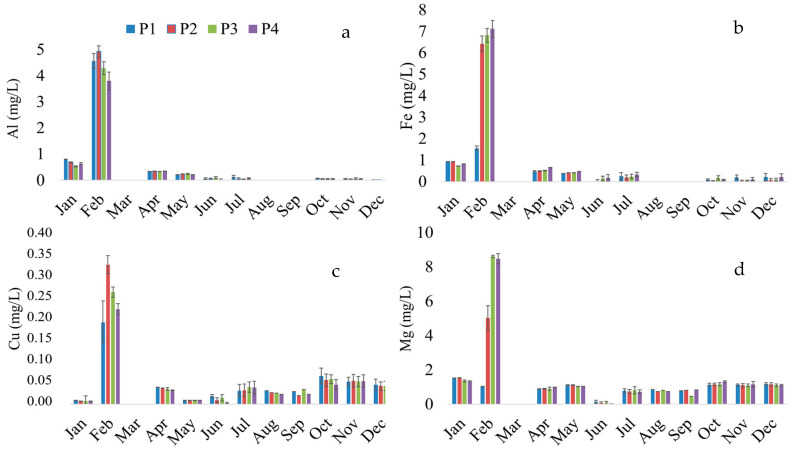
Temporal distribution of potentially toxic and essential elements in the waters of the middle Tocantins River throughout 2023, Maranhão, Brazil. (**a**) Aluminium (Al); (**b**) iron (Fe); (**c**) cooper (Cu); (**d**) magnesium (Mg). P1: Beira Rio (urban area), P2: Bananal (rural area), P3: Embiral (rural area), P4: Cidelândia (rural area). Rainiest/coldest (January to June) and hot/dry (July to December) seasons [39].

February exhibited elevated levels of Al (Figure 2a), Fe (Figure 2b), Cu (Figure 2c), and Mg (Figure 2d) in the water of the middle Tocantins River. This month falls within the rainy and cooler period (January to June). Interestingly, aluminum and iron were undetectable (<0.01 mg/L) in March, August, and September. This seasonal fluctuation indicates a complex dynamic influenced by climatic or seasonal factors. Additionally, copper and magnesium remained below the detection limit only in March. 

The rains began weakly in January; February experienced heavy rain and thunderstorms interspersed with sunny days, while in March, the rain was heavy and constant [74]. These variations highlight the need for further investigation to elucidate temporal patterns and potential influencing factors related to these elements in the middle Tocantins River waters. The results of potentially toxic and essential elements for the year 2023 are available in the Supplementary Materials (Tables S12–S24).

Studies have investigated the influence of natural factors, including seasonal variations, on the pollution of potentially toxic elements in surface water, yielding diverse findings [25,75,76]. Factors such as changes in water velocity, storage capacity, and spatial location can contribute to significant variations in the pollution levels of potentially toxic elements in water bodies [77]. 

Among the potentially toxic elements, only aluminum (Al) presented annual averages that exceeded national and international standards for water quality (P1: 0.69 mg/L; P2: 0.71 mg/L; P3: 0.63 mg/L; P4: 0.58 mg/L). Regarding essential elements, copper (Cu), iron (Fe), manganese (Mn), and selenium (Se) concentrations raise significant concerns for environmental and health quality (Table 4). No statistical differences were observed in the average concentrations among the studied sections of the middle Tocantins River.

The chemicals released from industrial sources may contaminate drinking water, either through direct discharges or indirectly, through widespread sources resulting from the use and disposal of materials and products containing these chemicals. Various chemicals may find their way into water bodies due to improper disposal of household or industrial products. Specifically, potentially toxic elements can be identified in domestic wastewater [78,79,80].

Aluminum (Al) is commonly found in food, drinking water, and antacid preparations, posing acute toxicity risks to humans upon oral ingestion. Exposure to aluminum has been linked to the development or acceleration of Alzheimer’s disease and various other health issues affecting nervous, reproductive, respiratory, mammary, skeletal, and immune tissues [49,81,82,83]. Health risk assessments for aluminum should consider individual factors such as age, renal function, diet, and gastric pH [84]. Elevated aluminum levels in brain tissues have been associated with encephalopathy, particularly notable in dialysis patients [85].

Copper (Cu) is an essential element for maintaining body metabolism but becomes toxic when present in higher concentrations [73]. Each metal, metalloid, and non-metal exhibits specific characteristics and toxicity. Its toxicity manifests as gastrointestinal irritation, particularly from drinking water contamination [49]. Furthermore, exposure to excessive levels of copper can lead to liver and kidney damage, anemia, immunotoxicity, and developmental toxicity [86].

The Tocantins–Araguaia region hosts the two largest iron ore deposits globally: the Carajás mine in Pará State (with 17 billion tons) and the Serra do Carmo iron deposit in Tocantins State (with 159 billion tons) [33]. Continuous monitoring of this water resource is essential for addressing and mitigating water pollution, as contaminated water poses serious health risks to human and animal health. Elements in drinking water such as fluoride, copper, zinc, or iron may exacerbate cognitive impairment or modify the neurotoxic effects of aluminum [87]. Selenium (Se) is also concerning due to its presence in the aquatic food chain, where it bioaccumulates in higher life forms [88].

### 3.3. Weighted Arithmetic Water Quality Index (WAWQI), Water Quality Index (WQI), and Quantitative Health Risk Assessment 

In this study, the WAWQI values were calculated using Equation (1) for four different locations in the middle Tocantins River: 7.42 (P1), 7.52 (P2), 8.27 (P3), and 8.65 (P4) (Table 5). According to the established classification criteria, where values between 0 and 25 are considered “Excellent,” the water quality in the middle Tocantins River can be categorized as such. The accessibility of safe drinking water is highlighted in this context, contributing significantly to various aspects of public health and well-being. This includes promoting healthy bodies, ensuring food security, reducing poverty, and fostering the overall development of a population, both socially and economically [89,90].

It is important to note that different studies or water quality assessment methodologies may have slightly different categorizations. Therefore, for comparison, the Water Quality Index (WQI) was applied using the national parameters [48]. The WQI values express freshwater quality as a percentage of an optimum situation, with 100% representing the best possible quality. The values were calculated using Equation (4) for four different locations in the Middle Tocantins River: 83.37 (P1), 83.75 (P2), 84.82 (P3), and 84.81 (P4) (Table 5). This indicates that all the analyzed sections are classified as “Good”.

The concentrations of Al, Cu, Fe, Mg, and Se through ingestion and dermal pathways (primarily through activities such as swimming and bathing in untreated water) for both adults and children are detailed in Table S25. Direct ingestion and dermal absorption (excluding inhalation through the mouth and nose) are recognized as the primary exposure routes for trace elements in river water for humans [91]. Importantly, the human health risk assessment values for both adults and children were found to be less than 1, indicating no risk.

Environmental exposure to metals through water may raise concerns regarding human exposure to potentially toxic elements [60]. Despite the studied elements showing average levels above national and international standards, human health risk assessment indices were found to be less than 1, indicating no risk (Table S25). This index assesses the probability of an individual developing cancer over their lifespan due to exposure to carcinogenic metal(loid)s. However, in this study, carcinogenic elements were not detected.

### 3.4. Correlation Coefficients and Principal Component Analysis (PCA)

The highlighted correlations reveal strong linear relationships: conductivity/TDS (r = 0.999), Al/Ca (r = 0.989), Ca/Si (r = 0.997), conductivity/NH_4_^+^ (r = 0.996), salinity/TDS (r = 0.993), Al/In (r = 0.989), and Fe/Ca (r = 0.947) (Table S26). Conversely, significant negative correlations were observed, such as Al/Na (r = −0.992), Fe/NO_2_ (r = −0.992), K/Se (r = −0.989), and chlorophyll/ORP (r = −0.966). High positive correlations (close to 1) imply a strong linear relationship, indicating that when one concentration increases, the other is expected to increase as well. Conversely, negative correlations suggest an inverse relationship between the variables. For instance, a negative correlation between Al/Na indicates that an increase in Al concentration is associated with a decrease in Na concentration. 

The correlation coefficients offer valuable insights into the strength and direction of relationships among the analyzed parameters, enhancing our understanding of water quality dynamics and potential contaminant sources [92]. Therefore, numerous studies employ both univariate and multivariate statistical analyses to identify highly correlated water pollutants and relevant industries [93,94]. This enables researchers to effectively trace these pollutants and industries, resulting in the development of more efficient pollution management strategies.

**Figure 3:**
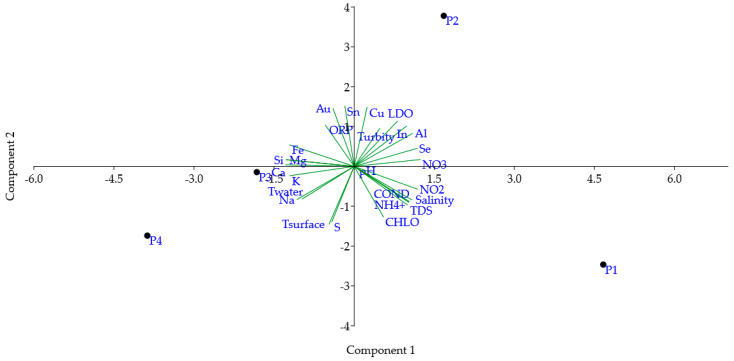
Principal component analysis (PCA) of water quality monitoring data of the middle Tocantins River, Maranhão, Brazil. P1: Beira Rio, P2: Bananal, P3: Embiral, P4: Cidelândia. T: temperature, TDS: total dissolved solids, LDO: luminescent dissolved oxygen. ORP: oxidation reduction potential. CHLO: chlorophyll. NH_4_^+^: ammonium, NO_2_: nitrite, and NO_3_^−^: nitrate.

The PCA results show that the first two components explain 89% of the data variability (Figure 3). The first component accounted for 52.79% (variance) and was positively loaded by conductivity, salinity, TDS, NH_4_^+^, NO_2_, NO_3_^−^, Al, and Se; this component presented negative loadings for Ca, Fe, K, Mg, Na, and Si. Conversely, the second component accounted for 35.88% (variance) and was positively loaded by LDO, Au, In, Cu, and Sn, while presenting negative loadings for T surface (air temperature), chlorophyll, and S. The second graph showing PC1 vs. PC3, along with the loadings, scores, and eigenvalues of the principal component analysis are available in the Supplementary Materials (Figure S2 and Tables S27–S29). 

PCA serves as a valuable tool for identifying pollution sources and extracting meaningful insights to facilitate eco-conservation and management efforts [94,95,96]. In this study, PCA analysis revealed three distinct groups based on water quality. The first group consisted solely of the P1 station, located within an urban area known as “Beira Rio,” which showed a positive association with the first component and a negative association with the second. 

The second group comprised only the P2 station, situated near the village of “Bananal” in a rural area, and exhibited a negative association with the second component of PCA. The third group consisted of the P3 and P4 stations, also situated in rural areas, being close to industrial waste discharge sites (pulp and paper production), and both stations showed a negative association with both the first and the second components. This underscores the utility of PCA in identifying pollution sources and guiding conservation and management efforts.

## 4. Conclusions

The levels of aluminum, copper, iron, magnesium, and selenium exceeded legal standards during 2023. The seasonal fluctuation indicates a complex dynamic influenced by climatic or seasonal factors, with February showing the highest values. Particularly, in urban areas such as P1, there are increased levels of conductivity, TDS, and chlorophyll, indicating the need for continuous monitoring. The presence of nitrogen compounds at P1 raises specific concerns regarding drinking water quality, which is a concern for the residents of the region, who use untreated river water for recreational and domestic purposes.

Despite seasonal fluctuations in element concentrations, the overall Water Quality Index (WAWQI) categorizes all sections as “Excellent,” while the Water Quality Index (WQI) is labeled as “Good.” This emphasizes the importance of ensuring safe drinking water for both public health and socioeconomic development. Human health risk assessments for aluminum, copper, iron, magnesium, and selenium indicate no risk, but ongoing monitoring and interventions are essential to maintain sustained water quality in the region.

## Figures and Tables

**Table 1:** Values for comparison of physicochemical parameters, nitrogenous compounds, and elements in the waters of the middle Tocantins River, Maranhão, Brazil.

Parameters	Standards for Drinking Water		
	CONAMA	WHO	USEPA
T Air (°C)	<30 °C	-	-
pH	6.0–9.0	6.5–8.5	-
Conductivity (µS/cm)	100	-	-
Salinity (PPT)	0.01	-	-
TDS (g/L)	0.5	0.3–0.9	-
LDO (mg/L)	≥6.0	-	-
Turbidity (NTU)	≤40	5	-
Chlorophyll (µG/L)	10	-	-
NH_4_^+^ (mg/L)	3.7 (pH < 7.5); 2.0 (7.5–8); 1.0 (8–8.5); 0.5 (pH > 8.5)	1.24	-
NO_2_ (mg/L)	1	0.1	-
NO_3_^−^ (mg/L)	10	10	-
Al	0.1	0.1–0.2	-
Hg	0.002	0.006	0.002
Cu	0.009	2	0.013
Fe	0.3	0.3	-
Mg	0.1	0.1	-
Na	-	200	-
Se	0.01	0.01	0.290

T: temperature, TDS: total dissolved solids, LDO: luminescent dissolved oxygen, NTU: nephelometric turbidity units. NH_4_^+^: ammonium, NO_2_: nitrite, and NO_3_^−^: nitrate. CONAMA: National Environmental Council Resolution n° 357/2005 [48]. WHO: World Health Organization [49]. USEPA: Environmental Protection Agency [50].

**Table 2:** Health risk assessment of different exposures through parameters.

Parameter	Children	Adults
Exposure Frequency (EF) (Day/year)	365	365
Body Weight (BW) (kg)	15	70
Ingestion Rate (IR) or Daily intake (DI) (L/day)	1.8	2.2
Exposure Duration (ED) (Years)	6	70
Skin Surface Area (SA) (cm^3^)	6600	18,000
Exposure Time (ET) (Hours/day)	1	0.58
Conversion Factor (CF) (L/cm^3^)	0.001	0.001
Averaging Time (AT) (Days)	365 × 6	365 × 70
Particular Emission Factor (PEM) (m^3^/kg)	1.3 × 10^9^	1.3 × 10^3^

Source: USEPA [58,59].

**Table 3:** Physicochemical parameter values and presence of nitrogen compounds in the middle Tocantins River waters during 2023, Maranhão, Brazil.

Parameters		Middle Tocantins River			
		P1	P2	P3	P4
T air (°C)	Range	26.5–32.2	25.5–30.3	24.5–33.1	25.7–32.0
	Mean ± SD	28.8 ± 1.7 ^a^	27.8 ± 1.4 ^a^	28.7 ± 2.1 ^a^	29.2 ± 1.8 ^a^
T water (°C)	Range	27.8–31.3	27.8–30.8	27.8–31.3	28.1–32.6
	Mean ± SD	29.3 ± 0.9 ^a^	29.2 ± 0.9 ^a^	29.4 ± 1.1 ^a^	29.9 ± 1.5 ^a^
pH	Range	4.25–9.19	5.38–9.5	5.48–9.52	4.81–9.01
	Mean ± SD	7.23 ± 1.03 ^a^	7.18 ± 0.67 ^a^	7.41 ± 1.04 ^a^	7.00 ± 1.03 ^a^
Conductivity (µS/cm)	Range	13.3–193.6	22.9–51.3	24–49	24.9–78.5
	Mean ± SD	51.67 ± 34 ^a^	37.70 ± 8.24 ^b^	36.93 ± 9.19 ^b^	39.68 ± 10.87 ^b^
Salinity (PPT)	Range	0.01–0.02	0–0.01	0–0.01	0–0.01
	Mean ± SD	* 0.02 ± 0.02 ^a^	0.01 ± 0.01 ^a^	0.01 ± 0.01 ^a^	0.01 ± 0.01 ^a^
TDS (g/L)	Range	0.1002–0.0471	0.0144–0.0328	0.0100–0.0318	0.0060–0.0502
	Mean ± SD	0.0326 ± 0.02 ^a^	0.0242 ± 0.01 ^b^	0.0239 ± 0.01 ^b^	0.0251 ± 0.01 ^b^
LDO (mg/L)	Range	7.56–13	8.53–13.68	7.64–13.42	6.71–13.28
	Mean ± SD	8.91 ± 3.25 ^a^	9.36 ± 3.33 ^a^	8.74 ± 3.14 ^a^	8.77 ± 3.10 ^a^
Turbidity (NTU)	Range	18.5–117.7	21.6–113.8	23.7–123.7	24.6–114.5
	Mean ± SD	49.56 ± 24.34 ^a^	45.04 ± 23.55 ^a^	40.92 ± 28.25 ^a^	42.84 ± 28.63 ^a^
Chlorophyll (µG/L)	Range	0.52–3.7	0.41–2.8	0.52–2.84	0.47–2.7
	Mean ± SD	1.47 ± 0.95 ^a^	1.24 ± 0.80 ^b^	1.35 ± 0.75 ^a^	1.28 ± 0.80 ^ab^
ORP (mV)	Range	5–297	18–273	21–195	20–380
	Mean ± SD	123 ± 63.22 ^a^	152 ± 132.85 ^a^	131 ± 38.40 ^a^	150 ± 75.41 ^a^
NH_4_^+^ (mg/L)	Range	1.55–12.82	<0.01–4.97	<0.01–3.66	<0.01–6.2
	Mean ± SD	* 3.96 ± 2.46 ^a^	* 1.59 ± 1.13 ^b^	* 1.65 ± 0.97 ^b^	* 1.91 ± 1.64 ^b^
NO_2_ (mg/L)	Range	<0.01–0.43	<0.01–0.08	<0.01–0.06	<0.01–0.07
	Mean ± SD	* 0.07 ± 0.10 ^a^	0.03 ± 0.03 ^b^	0.02 ± 0.02 ^b^	0.02 ± 0.02 ^b^
NO_3_^−^ (mg/L)	Range	<0.01–6.23	<0.01–3.86	<0.01–3.44	<0.01–3.48
	Mean ± SD	0.98 ± 1.59 ^a^	0.86 ± 1.28 ^a^	0.60 ± 1.14 ^a^	0.60 ± 1.04 ^a^

P1: Beira Rio (urban area), P2: Bananal (rural area), P3: Embiral (rural area), P4: Cidelândia (rural area). T: temperature, TDS: total dissolved solids, LDO: luminescent dissolved oxygen, NTU: nephelometric turbidity units, ORP: oxidation reduction potential. NH_4_^+^: ammonium, NO_2_: nitrite, and NO_3_^−^: nitrate. * Parameter concentrations above the quality standards of some regulations in comparison. The Tukey test was performed (*p* < 0.05) for mean comparison, where “^a^” and “^b^” alone denote significant differences among other groups.

**Table 4:** Comparison of the concentration of potentially toxic and essential elements (annual average, mg/L) in water from the middle Tocantins River with national and international regulations.

Elements (Detection Limit)	Tocantins River			
	P1	P2	P3	P4
Potentially toxic elements				
Al (0.01)	* 0.69 ± 1.42	* 0.71 ± 1.54	* 0.63 ± 1.34	* 0.58 ± 1.19
Au (0.01)	0.08 ± 0.16	0.11 ± 0.24	0.10 ± 0.20	0.09 ± 0.19
Hg (0.01)	0.0002 ± 0	0.0002 ± 0	0.0002 ± 0	0.0002 ± 0
In (0.03)	0.58 ± 1.05	0.64 ± 1.20	0.51 ± 0.89	0.44 ± 0.80
Essential elements				
Ca (0.01)	8.24 ± 3.38	10.78 ± 8.98	12.59 ± 17.47	13.89 ± 17.88
Cu (0.02)	* 0.05 ± 0.05	* 0.06 ± 0.09	* 0.05 ± 0.07	* 0.05 ± 0.06
Fe (0.02)	* 0.46 ± 0.49	* 0.95 ± 2.00	* 1.02 ± 2.11	* 1.11 ± 2.19
K (0.02)	0.48 ± 0.40	0.59 ± 0.85	0.65 ± 0.89	0.87 ± 1.77
Mg (0.02)	* 0.96 ± 0.35	* 1.31 ± 1.29	* 1.59 ± 2.28	* 1.61 ± 2.23
Na (0.02)	0.92 ± 0.75	0.83 ± 0.83	1.14 ± 1.37	1.45 ± 1.72
S (0.01)	0.17 ± 0.16	0.09 ± 0.11	0.14 ± 0.15	0.21 ± 0.28
Se (0.02)	* 0.20 ± 0.01	* 0.19	* 0.17 ± 0.02	* 0.13 ± 0.01
Si (0.01)	4.46 ± 2.16	6.11 ± 7.16	6.96 ± 9.66	7.61 ± 9.71
Sn (0.02)	0.27 ± 0.50	0.39 ± 0.80	0.34 ± 0.66	0.29 ± 0.60

P1: Beira Rio (urban area), P2: Bananal (rural area), P3: Embiral (rural area), P4: Cidelândia (rural area). * Parameter concentrations above the quality standards of some regulations for comparison.

**Table 5:** The WAWQI and WQI estimates for surface water parameters with arithmetic weights in the middle Tocantins River, Maranhão, Brazil.

Index	Tocantins River			
	P1	P2	P3	P4
WAWQI	7.42	7.52	8.27	8.65
WQI	83.37	83.75	84.82	84.81

P1: Beira Rio (urban area), P2: Bananal (rural area), P3: Embiral (rural area), P4: Cidelândia (rural area). Weighted Arithmetic Water Quality Index (WAWQI) [45,46]. WQI: Water Quality Index. All physicochemical parameters are expressed in mg/L except temperature (water temperature °C) and pH.

## Data Availability

The datasets generated during the current study are available in the Supplementary Materials.

## Supplementary Materials

The following supporting information can be downloaded at: https://www.mdpi.com/article/10.3390/toxics12070444/s1, Figure S1: Climatology and weather forecast history of 2023 in Imperatriz, Brazil; Figure S2: Principal component analysis (PC1 vs. PC3) of water quality monitoring data of the middle Tocantins River, Maranhão, Brazil; Table S1: Temperature (air and water) of the middle Tocantins River throughout 2023; Table S2: pH (unit) in the waters of the middle Tocantins River throughout 2023; Table S3: Conductivity (mS/cm) in the waters of the middle Tocantins River throughout 2023; Table S4: Salinity (PPT) in the waters of the middle Tocantins River throughout 2023; Table S5: Total dissolved solids (g/L) in the waters of the middle Tocantins River throughout 2023; Table S6: Luminescent dissolved oxygen (mg/L) in the waters of the middle Tocantins River throughout 2023; Table S7: Turbidity (NTU) in the waters of the middle Tocantins River throughout 2023; Table S8: Chlorophyll (µG/L) in the waters of the middle Tocantins River throughout 2023; Table S9: ORP (mV) in the waters of the middle Tocantins River throughout 2023; Table S10: Ammonium (NH_4_^+^) (mg/L) in the waters of the middle Tocantins River throughout 2023; Table S11: Nitrite (NO_2_) (mg/L) in the waters of the middle Tocantins River throughout 2023; Table S12: Nitrate (NO_3_^−^) (mg/L) in the waters of the middle Tocantins River throughout 2023; Table S13: Results of essential and potentially toxic elements in the waters of the middle Tocantins River throughout 2023; Table S14: Results of essential and potentially toxic elements in the waters of the middle Tocantins River throughout 2023; Table S15: Results of essential and potentially toxic elements in the waters of the middle Tocantins River throughout 2023; Table S16: Results of essential and potentially toxic elements in the waters of the middle Tocantins River throughout 2023; Table S17: Results of essential and potentially toxic elements in the waters of the middle Tocantins River throughout 2023; Table S18: Results of essential and potentially toxic elements in the waters of the middle Tocantins River throughout 2023; Table S19: Results of essential and potentially toxic elements in the waters of the middle Tocantins River throughout 2023; Table S20: Results of essential and potentially toxic elements in the waters of the middle Tocantins River throughout 2023; Table S21: Results of essential and potentially toxic elements in the waters of the middle Tocantins River throughout 2023; Table S22: Results of essential and potentially toxic elements in the waters of the middle Tocantins River throughout 2023; Table S23: Results of essential and potentially toxic elements in the waters of the middle Tocantins River throughout 2023; Table S24: Results of essential and potentially toxic elements in the waters of the middle Tocantins River throughout 2023; Table S25: The human health risk assessment indices for cancer risks from ingestion and absorption of studied metals for children and adults; Table S26: Pearson correlation coefficients for water quality monitoring data of the middle Tocantins River, Maranhão, Brazil; Table S27: Loadings from principal component analysis (PCA); Table S28: Scores from principal component analysis (PCA); Table S29: Summary from principal component analysis (PCA).
