# Supplementary material for: Levels of Potentially Toxic and Essential Elements in Water and Estimation of Human Health Risks in a River Located at the Interface of Brazilian Savanna and Amazon Biomes (Tocantins River)
Source: Toxics. 2024 Jun 21;12(7):444. doi: 10.3390/toxics12070444 (PMC11280896; doi:10.3390/toxics12070444)
Supplement: Supplementary file 1 [file toxics-12-00444-s001.zip › toxics-3047015-supplementary.pdf]

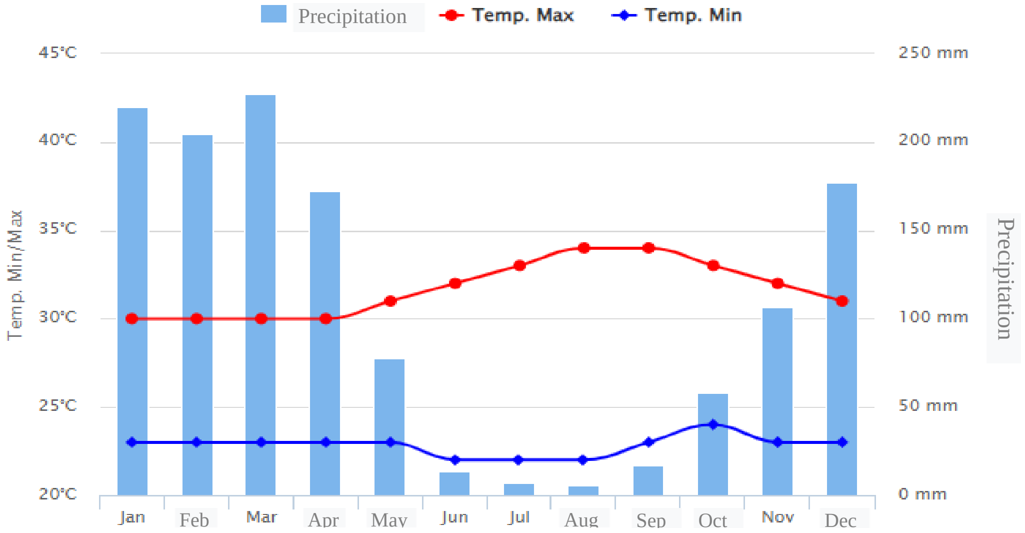

Figure S1. Climatology and weather forecast history of 2023 in Imperatriz, Brazil. Source: Climatempo (2023).

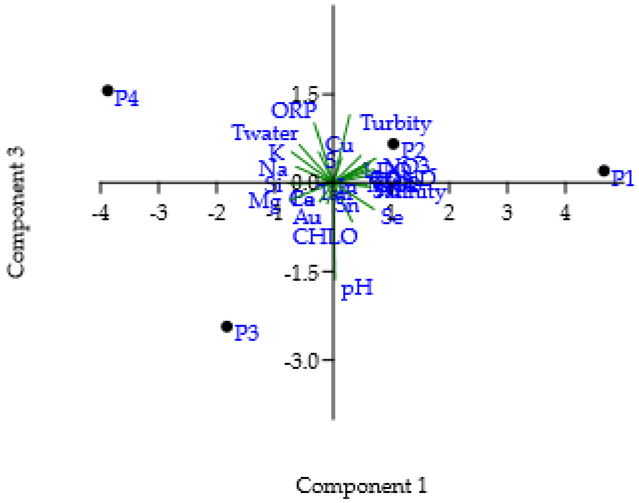

Figure S2. Principal component analysis (PC1 vs PC3) of water quality monitoring data of the middle Tocantins River, Maranhão, Brazil.

Table S1. Temperature (air and water) of the middle Tocantins River throughout 2023.

Sampling area		Temperature (ºC) - 2023													
Sampling area		January	February	March	April	May	June	July	August	September	October	November	December	Average	Standard deviation
P1	Air	30.7	32.2	30.3	27.5	27	26.5	27.7	29.3	30.3	27.9	28	28.2	28.8	1.7
	Water	28.7	30	29.3	29.1	29	27.8	28.6	29.6	31.3	29.5	29.6	29	29.3	0.9
P2	Air	28	28.8	26.6	25.5	29.2	28.5	26.5	28.7	30.3	28.1	26.6	26.4	27.8	1.4

	Water	27.8	28.3	28.8	28.1	29.7	29.3	28.9	29.5	30.8	29.8	29.9	29.5	29.2	0.9
P3	Air	29.6	30.3	24.5	29.3	26.5	28	28.3	28.5	30.2	27.3	33.1	28.4	28.7	2.1
	Water	28.3	28.3	27.8	28.9	28.6	29.3	28.9	29.7	31.2	30	31.3	29.9	29.4	1.1
P4	Air	28.1	28.2	28.5	25.7	29.1	27	30.5	30.6	30.3	29.7	32	31.2	29.2	1.8
	Water	28.3	28.3	28.7	28.1	29.5	29.3	31.2	31.2	31.4	30.6	32.6	30.1	29.9	1.5

P1: Beira Rio (urban area), P2: Bananal (rural area), P3: Embiral (rural area), P4: Cidelândia (rural area).

Table S2. pH (Unit) in the waters of the middle Tocantins River throughout 2023.

pH (Unit) - 2023																
Sampling area		January	February	March	April	May	June		July	August	September	October	November	December	Average	Standard deviation
P1	BR1	7.17	7.05	7.24	7.30	4.25	5.88	6.48	6.48	9.19	8.7	8.78	6.87	7.12	7.23	1.03
	BR2	7.20	7.41	7.22	7.41	6.57	6.03	6.64	6.64	8	8.82	7.67	7.78	7.28		
	BR3	7.17	7.39	7.24	7.42	6.98	5.29	6.40	6.40	8.5	8.14	8.05	8.63	7.30		
P2	B1	7.16	6.89	7.26	7.27	6.67	6.83	7.41	7.41	8.29	7.42	6.55	5.84	7.08	7.18	0.67
	B2	7.10	7.62	7.27	7.00	7.00	6.98	6.98	6.98	7.52	7.52	6.34	7.5	7.15		
	B3	7.11	7.70	7.29	7.32	7.13	6.98	6.78	6.78	9.5	8.14	5.38	7.57	7.31		
P3	E1	5.48	7.67	7.11	7.39	6.33	6.98	6.48	6.48	8.68	8.13	9	8.55	7.36	7.41	
	E2	5.40	7.61	7.16	7.23	6.76	7.06	6.51	6.51	8.01	8.52	9.13	9.08	7.42		
	E3	6.00	7.58	7.20	7.41	7.00	6.73	6.51	6.51	8.31	8.38	9.52	8.27	7.45		
P4	V1	5.76	7.68	6.45	7.81	4.81	6.48	6.38	6.38	8.53	8.6	5.26	7.03	6.76	7.00	1.03
	V2	5.80	7.40	6.84	7.44	6.24	6.42	6.22	6.43	9.01	8.11	7.48	8.15	7.13		
	V3	5.88	6.26	7.06	7.37	6.27	6.49	6.87	6.47	8.57	8.39	7.15	8.65	7.12		

P1: Beira Rio (urban area), P2: Bananal (rural area), P3: Embiral (rural area), P4: Cidelândia (rural area).

Table S3. Conductivity (µS/cm) in the waters of the middle Tocantins River throughout 2023.

Conductivity (µS/cm) - 2023																
Sampling area		January	February	March	April	May	June	July	August	September	October	November	December	Average		Standard deviation
	BR1	55.2	193.6	53.0	67.1	44.2	22.2	42.0	41.4	31.6	28.3	34.4	48.3	55.11	51.67	34.00
P1	BR2	55.0	65.5	55.2	64.9	40.8	22.2	52.2	156.3	37.1	30.9	30.6	35.6	53.86		
	BR3	56.0	63.8	73.8	71.6	28.2	13.3	48.6	47.9	44.4	31.5	34.7	38.8	46.05		
P2	B1	38.7	51.3	43.8	49.5	41.5	38.8	37.2	36.9	22.8	27.5	43.4	55.9	40.61	37.70	8.24
	B2	40.0	39.0	43.6	49.8	40.8	39.0	35.0	38.0	23	28.6	28.3	30.4	36.29		
	B3	41.0	40.6	43.4	49.8	40.9	39.0	33.5	35.0	22.9	28.5	29.3	30.4	36.19		
P3	E1	47.8	48.7	44.0	49.4	38.4	15.7	37.6	33.3	23.9	23.9	31.7	31.3	35.48	36.93	9.19
	E2	48.0	48.2	43.8	48.9	30.5	40.6	38.5	39.9	24	25.5	33.1	31.1	37.68		
	E3	48.1	47.2	43.9	49.0	30.5	38.9	39.0	39.7	24.4	28.1	31.8	31	37.63		
P4	V1	47.8	44.8	49.2	48.4	34.5	39.4	45.5	45.2	25.7	30	32.3	39	40.15	39.68	10.87
	V2	48.0	46.8	43.4	48.4	31.9	35.8	46.7	44.7	24.9	16.2	33.3	32.5	37.72		
	V3	47.8	78.5	43.6	47.8	28.8	41.4	45.9	40.0	25.1	30.7	32.4	32.1	41.18		

P1: Beira Rio (urban area), P2: Bananal (rural area), P3: Embiral (rural area), P4: Cidelândia (rural area).

Table S4. Salinity (PPT) in the waters of the middle Tocantins River throughout 2023.

Salinity (PPT) - 2023																
Sampling area		January	February	March	April	May	June	July	August	September	October	November	December	Average		Standard deviation
	BR1	0.01	0.09	0.01	0.03	0.01	0.01	0.01	0.01	0.03	0	0	0.01	0.02	0.02	0.02
P1	BR2	0.02	0.02	0.01	0.03	0.01	0.01	0.01	0.07	0.01	0	0	0	0.02		
	BR3	0.02	0.02	0.03	0.03	0.01	0.01	0.01	0.01	0.01	0	0	0.01	0.01		

P2	B1	0.01	0.01	0.01	0.01	0.01	0.01	0.00	0.00	0.00	0	0.01	0.01	0.01	0.01	0.01
	B2	0.01	0.01	0.01	0.01	0.01	0.01	0.00	0.00	0.00	0	0	0	0.01		
	B3	0.01	0.01	0.01	0.01	0.01	0.01	0.00	0.00	0.00	0	0	0	0.01		
P3	E1	0.01	0.01	0.01	0.01	0.01	0.01	0.00	0.00	0.00	0	0	0	0.01	0.01	0.01
	E2	0.01	0.01	0.01	0.01	0.01	0.01	0.01	0.00	0.00	0	0	0	0.01		
	E3	0.01	0.01	0.01	0.01	0.01	0.01	0.01	0.00	0.00	0	0	0	0.01		
P4	V1	0.01	0.01	0.02	0.02	0.01	0.01	0.01	0.01	0.00	0	0	0	0.01	0.01	0.01
	V2	0.01	0.01	0.01	0.01	0.01	0.01	0.01	0.01	0.00	0	0	0	0.01		
	V3	0.01	0.03	0.01	0.01	0.01	0.01	0.01	0.00	0.00	0	0	0	0.01		

P1: Beira Rio (urban area), P2: Bananal (rural area), P3: Embiral (rural area), P4: Cidelândia (rural area).

Table S5. Total Dissolved Solids (g/L) in the waters of the middle Tocantins River throughout 2023.

Total Dissolved Solids (TDS. G/ L) - 2023																
Sampling area		January	February	March	April	May	June	July	August	September	October	November	December	Average		Standard deviation
	BR1	0.0353	0.1239	0.0338	0.0431	0.0283	0.0040	0.0269	0.0265	0.0238	0.0181	0.022	0.0309	0.035	0.0326	0.02
P1	BR2	0.0355	0.0419	0.0353	0.0417	0.0261	0.0142	0.0334	0.1002	0.0222	0.0198	0.0196	0.0228	0.034		
	BR3	0.0340	0.0407	0.0471	0.0456	0.0180	0.0085	0.0311	0.0307	0.0202	0.0202	0.0222	0.0248	0.029		
P2	B1	0.0248	0.0328	0.0290	0.0318	0.0266	0.0248	0.0239	0.0236	0.0146	0.0176	0.0278	0.0358	0.026	0.0242	0.01
	B2	0.0255	0.0250	0.0280	0.0317	0.0261	0.0250	0.0224	0.0243	0.0147	0.0183	0.0181	0.0195	0.023		
	B3	0.0244	0.0260	0.0280	0.0317	0.0262	0.0250	0.0215	0.0243	0.0144	0.0182	0.0188	0.0194	0.023		
P3	E1	0.0306	0.0312	0.0280	0.0318	0.0246	0.0100	0.0240	0.0213	0.0153	0.0153	0.0203	0.02	0.023	0.0239	0.01
	E2	0.0300	0.0309	0.0281	0.0314	0.0264	0.0260	0.0247	0.0256	0.0153	0.0163	0.0212	0.0199	0.025		
	E3	0.0288	0.0303	0.0282	0.0313	0.0251	0.0249	0.0249	0.0254	0.0156	0.018	0.0204	0.0199	0.024		
P4	V1	0.0307	0.0287	0.0316	0.0313	0.0235	0.0060	0.0291	0.0290	0.0164	0.0192	0.0207	0.0249	0.024	0.0251	0.01
	V2	0.0300	0.0300	0.0279	0.0310	0.0278	0.0229	0.0299	0.0286	0.016	0.0104	0.0213	0.0208	0.025		
	V3	0.0311	0.0502	0.0281	0.0305	0.0276	0.0265	0.0294	0.0145	0.0161	0.0197	0.0207	0.0205	0.026		

P1: Beira Rio (urban area), P2: Bananal (rural area), P3: Embiral (rural area), P4: Cidelândia (rural area).

Table S6. Luminescent Dissolved Oxygen (mg/L) in the waters of the middle Tocantins River throughout 2023.

Luminescent Dissolved Oxygen (LDO. mg/L) - 2023																
Sampling area		January	February	March	April	May	June	July	August	September	October	November	December	Average		Standard deviation
	BR1	7.65	7.66	0.00	7.65	8.63	7.94	12.72	8.65	9.4	12.25	11.48	13	8.92	8.91	3.25
P1	BR2	7.90	9.57	0.00	7.63	8.46	8.56	11.96	8.52	9.76	11.44	11.65	12	8.95		
	BR3	7.99	10.49	0.00	7.56	8.44	8.64	10.30	10.00	7.23	11.97	12	11.81	8.87		
P2	B1	8.53	8.82	0.00	8.82	9.27	9.31	13.04	12.91	9.21	8.98	13.68	10.86	9.45	9.36	3.33
	B2	8.55	9.25	0.00	8.86	9.11	9.16	12.22	9.61	6.22	10.43	12.6	12.21	9.02		
	B3	8.67	9.33	0.00	8.86	9.41	9.53	11.41	12.96	10.81	10.46	13.37	10.66	9.62		
P3	E1	8.00	10.78	0.00	8.06	8.01	8.61	10.95	10.02	10.05	8.44	7.83	8.88	8.30	8.74	3.14
	E2	7.99	11.47	0.00	8.17	9.28	10.05	12.60	13.04	8.87	8.74	9.35	9.01	9.05		
	E3	8.10	11.33	0.00	8.35	8.58	9.27	13.10	13.42	9.15	10.53	7.71	6.88	8.87		
P4	V1	8.50	10.72	0.00	8.31	7.70	8.94	10.06	12.91	10.91	11.51	13.28	6.71	9.13	8.77	3.10
	V2	8.60	11.11	0.00	8.73	8.46	9.10	9.21	11.90	10	9.23	10.83	6.71	8.66		
	V3	8.44	10.03	0.00	8.70	8.32	9.82	8.85	10.24	10.01	11.77	8.96	7.08	8.52		

P1: Beira Rio (urban area), P2: Bananal (rural area), P3: Embiral (rural area), P4: Cidelândia (rural area).

Table S7. Turbidity (NTU) in the waters of the middle Tocantins River throughout 2023.

Turbidity (NTU) - 2023

Sampling area		January	February	March	April	May	June	July	August	September	October	November	December	Average		Standard deviation
	BR1	55.0	56.0	55.0	24.3	19.9	61.7	117.7	68.0	44.5	35.8	37.5	48.6	52.00	49.56	24.34
P1	BR2	58.0	59.0	54.9	24.4	19.1	34.6	115.3	55.0	39.5	35.5	37.3	48.6	48.43		
	BR3	59.0	60.0	54.9	24.5	18.5	29.4	114.7	50.9	46.3	35.3	37	48.6	48.26		
P2	B1	57.9	34.1	55.1	21.6	31.3	24.3	113.8	45.0	36	36.3	45.6	50	45.92	45.04	23.55
	B2	58.0	31.5	55.1	22.1	34.5	23.9	113.3	40.9	35.2	36.2	42.6	48.5	45.15		
	B3	58.1	35.1	55.6	22.7	25.8	24.1	112.7	37.6	34	36	41.5	45.5	44.06		
P3	E1	53.0	0.0	55.0	24.5	27.7	29.1	123.7	34.4	37	37.3	38.7	41	41.78	40.92	28.25
	E2	53.3	0.0	55.1	23.7	30.5	23.8	120.3	27.8	36.6	36.9	38.4	42	40.70		
	E3	53.2	0.0	55.1	24.1	30.5	25.5	119.0	21.9	36.4	36.6	38	43	40.28		
P4	V1	54.3	0.0	114.5	25.9	34.6	25.3	112.3	35.3	38.6	39.1	39.8	44	46.98	42.84	28.63
	V2	54.4	0.0	54.7	22.0	31.9	24.9	111.9	33.4	38	38.3	39.4	47	41.33		
	V3	55.0	0.0	55.0	21.2	28.8	24.6	111.8	32.0	37.4	37.8	39.1	40	40.23		

P1: Beira Rio (urban area), P2: Bananal (rural area), P3: Embiral (rural area), P4: Cidelândia (rural area).

Table S8. Chlorophyll (μG/ L) in the waters of the middle Tocantins River throughout 2023.

Chlorophyll (μG/ L) - 2023																
Sampling area		January	February	March	April	May	June	July	August	September	October	November	December	Average		Standard deviation
	BR1	2.70	3.70	2.26	2.00	1.87	0.70	0.60	0.52	1.05	0.61	0.78	0.7	1.46	1.47	0.95
P1	BR2	2.88	3.55	2.05	2.15	1.93	0.73	0.62	1.15	1.11	0.8	0.56	0.79	1.53		
	BR3	2.75	3.22	1.97	2.05	1.91	0.88	0.68	0.52	0.88	0.71	0.75	0.84	1.43		
	B1	2.80	2.32	1.98	1.70	1.57	0.70	0.64	0.46	0.97	0.69	0.44	0.47	1.23	1.24	0.80
P2	B2	2.79	2.31	1.74	1.62	1.57	0.86	0.62	0.41	0.8	0.64	0.62	0.54	1.21		
	B3	2.88	2.42	2.44	1.64	1.41	0.82	0.72	0.54	0.74	0.68	0.46	0.58	1.28		
	E1	2.00	2.84	1.99	1.87	2.05	0.86	0.59	0.48	1.09	0.49	0.52	0.59	1.28	1.35	0.75
P3	E2	2.01	2.78	2.02	2.09	1.81	1.31	0.97	0.56	0.8	0.71	0.56	0.52	1.35		
	E3	1.99	2.48	1.90	2.42	2.17	0.71	1.29	1.11	0.86	0.57	0.69	0.8	1.42		
	V1	2.00	2.07	2.15	2.20	1.87	0.66	0.70	0.86	0.76	0.38	0.66	0.69	1.25	1.28	0.80
P4	V2	2.02	2.11	2.49	2.26	2.27	0.63	0.66	0.47	0.74	0.5	0.55	0.54	1.27		
	V3	2.10	2.70	2.47	2.16	2.00	0.67	0.65	0.50	0.71	0.48	0.82	0.5	1.31		

P1: Beira Rio (urban area), P2: Bananal (rural area), P3: Embiral (rural area), P4: Cidelândia (rural area).

Table S9. ORP (mV) in the waters of the middle Tocantins River throughout 2023.

ORP (MV) - 2023																
Sampling area		January	February	March	April	May	June	July	August	September	October	November	December	Average		Standard deviation
	BR1	162	120	119	112	32	297	70	230	44	36	87	173	123.50	123	63.22
P1	BR2	160	117	123	116	5	172	117	261	105	56	88	177	124.75		
	BR3	158	115	119	115	24	131	78	204	100	109	137	158	120.67		
P2	B1	195	194	143	130	159	157	81	139	45	39	273	211	147.13	152	132.85
	B2	200	163	144	125	150	146	52	833	47	45	261	148	192.79		
	B3	197	158	146	131	158	148	18	25	65	53	165	135	116.60		
P3	E1	195	133	152	130	154	132	130	147	35	129	110	162	134.05	131	38.40
	E2	190	120	145	121	175	140	112	143	35	128	111	160	131.71		
	E3	189	110	143	126	167	137	99	139	21	122	120	141	126.10		
P4	V1	218	145	219	380	214	240	35	45	43	179	154	216	173.96	150	75.41
	V2	222	153	202	117	189	165	24	49	45	154	149	188	138.10		
	V3	220	152	154	130	170	152	20	143	46	139	140	176	136.75		

P1: Beira Rio (urban area), P2: Bananal (rural area), P3: Embiral (rural area), P4: Cidelândia (rural area).

Table S10. Ammonium (NH_4^+) (mg/L) in the waters of the middle Tocantins River throughout 2023.

Ammonium (NH_4^+. mg/L)										
Sampling area	April	May	June	July	August	September	October	November	December	Average	Standard deviation

	BR1	2.78	2.33	2.18	2.38	4.02	4.11	4.11	4.00	6.45	3.60	3.96	2.46
P1	BR2	1.55	4.70	6.45	2.94	12.82	3.19	3.19	2.02	3.19	4.45		
	BR3	1.88	4.29	1.32	1.59	8.16	3.66	3.64	6.86	3.14	3.84		
P2	B1	1.91	1.61	0.69	0.01	2.83	1.95	1.95	0.67	3.14	1.64	1.59	1.13
	B2	1.23	1.14	0.02	0.22	2.06	1.41	1.41	1.73	3.01	1.36		
	B3	0.44	4.97	0.06	1.34	1.61	1.16	1.16	2.15	2.96	1.76		
P3	E1	1.43	2.78	0.49	2.38	1.95	2.06	3.66	2.74	1.84	2.15	1.65	0.97
	E2	0.71	1.19	0.20	0.01	1.86	2.94	2.94	1.52	1.30	1.41		
	E3	0.31	1.16	0.02	2.06	1.61	2.29	2.29	0.98	1.70	1.38		
	V1	0.92	3.39	1.41	1.16	4.52	2.36	2.36	2.67	2.20	2.33	1.91	1.64
P4	V2	1.61	6.20	0.24	1.19	3.91	1.34	1.34	1.19	0.87	1.99		
	V3	1.52	6.16	0.01	0.01	1.41	0.94	0.94	0.85	0.85	1.41		

P1: Beira Rio (urban area), P2: Bananal (rural area), P3: Embiral (rural area), P4: Cidelândia (rural area).

Table S11. Nitrite (NO^2) (mg/L) in the waters of the middle Tocantins River throughout 2023.

Nitrite (NO^2) (mg/L)													
Sampling area		April	May	June	July	August	September	October	November	December	Average		Standard deviation
	BR1	0.01	0.01	0.01	0.01	0.10	0.10	0.10	0.01	0.01	0.04	0.07	0.10
P1	BR2	0.01	0.01	0.01	0.01	0.07	0.11	0.11	0.37	0.00	0.08		
	BR3	0.02	0.07	0.03	0.05	0.09	0.09	0.09	0.43	0.00	0.10		
P2	B1	0.01	0.00	0.01	0.01	0.01	0.08	0.08	0.01	0.01	0.03	0.03	0.03
	B2	0.01	0.01	0.01	0.01	0.01	0.10	0.10	0.01	0.01	0.03		
	B3	0.01	0.01	0.01	0.01	0.01	0.08	0.08	0.01	0.01	0.03		
P3	E1	0.02	0.01	0.01	0.01	0.01	0.06	0.06	0.01	0.01	0.02	0.02	0.02
	E2	0.01	0.01	0.01	0.01	0.01	0.06	0.06	0.01	0.01	0.02		
	E3	0.01	0.01	0.01	0.01	0.01	0.06	0.06	0.01	0.00	0.02		
P4	V1	0.01	0.01	0.01	0.01	0.01	0.07	0.07	0.01	0.01	0.02	0.02	0.02
	V2	0.01	0.01	0.01	0.01	0.01	0.07	0.07	0.01	0.01	0.02		
	V3	0.00	0.03	0.01	0.01	0.01	0.06	0.06	0.01	0.01	0.02		

P1: Beira Rio (urban area), P2: Bananal (rural area), P3: Embiral (rural area), P4: Cidelândia (rural area).

Table S12. Nitrate (NO_3^-) (mg/L) in the waters of the middle Tocantins River throughout 2023.

Nitrate (NO_3^-) (mg/L)													
Sampling area		April	May	June	July	August	September	October	November	December	Average		Standard deviation
	BR1	0.01	0.074	0.01	0.01	6.227	0.107	0.242	2.649	3.572	1.43	0.98	1.59
P1	BR2	0.01	0.105	0.01	0.01	0.010	0.242	0.242	2.222	3.414	0.70		
	BR3	0.010	0.033	0.010	0.010	2.514	0.062	0.062	2.222	2.244	0.80		
	B1	0.01	0.093	0.01	0.01	0.01	0.010	0.010	2.109	2.379	0.52	0.86	1.28
P2	B2	0.01	0.022	0.01	0.01	0.01	1.389	1.389	3.842	3.257	1.10		
	B3	0.01	0.044	0.01	0.01	0.01	1.164	1.164	2.379	3.864	0.96		
	E1	0.01	0.143	0.01	0.01	0.01	0.010	0.010	3.324	2.267	0.64	0.60	1.14
P3	E2	0.01	0.134	0.01	0.01	0.01	0.010	0.010	3.437	1.974	0.62		
	E3	0.01	0.050	0.01	0.01	0.01	0.010	0.010	2.267	2.537	0.55		
	V1	0.01	0.043	0.01	0.01	0.01	0.062	0.377	3.482	2.109	0.68	0.60	1.04
P4	V2	0.01	0.201	0.01	0.01	0.01	0.377	0.354	2.694	2.087	0.64		
	V3	0.01	0.014	0.01	0.01	0.01	0.010	0.264	2.649	1.479	0.50		

P1: Beira Rio (urban area), P2: Bananal (rural area), P3: Embiral (rural area), P4: Cidelândia (rural area).

Table S13. Results of essential and potentially toxic elements in the waters of the middle Tocantins River throughout 2023.

Elements (mg/L) - January. 2023												
Sampling area		**Al**	**Au**	**Ca**	**Cu**	**Fe**	**K**	**Mg**	**Na**	**S**	**Si**	**Sn**
	BR1	0.784	0.012	12.7	0.0083	0.938	0.43	1.53	0.601	0.0706	5.84	0.0236
P1	BR2	0.801	0.012	12.5	0.0083	0.932	0.43	1.53	0.602	0.0706	5.9	0.0236
	BR3	0.795	0.014	12.6	0.009	0.933	0.42	1.52	0.6	0.077	5.99	0.022
	B1	0.697	0.0125	12.6	0.0079	0.933	0.4	1.56	0.51	0.0412	5.91	0.0219
P2	B2	0.666	0.0125	12	0.0079	0.933	0.4	1.55	0.51	0.0412	5.92	0.0219
	B3	0.687	0.013	12.5	0.0078	0.922	0.42	1.56	0.53	0.044	5.93	0.02
	E1	0.525	0.0124	11.8	0.0081	0.736	0.51	1.4	0.537	0.0576	5.47	0.0179
P3	E2	0.499	0.012	11.8	0.0081	0.736	0.51	1.4	0.54	0.0576	5.47	0.0179
	E3	0.555	0.013	11.7	0.007	0.72	0.55	1.3	0.52	0.06	5.4	0.016
	V1	0.606	0.0104	11.3	0.0077	0.836	0.409	1.35	0.615	0.0518	5.59	0.0102
P4	V2	0.609	0.0104	11	0.0077	0.83	0.41	1.35	0.6	0.0518	5.66	0.01
	V3	0.671	0.1	11.2	0.008	0.82	0.42	1.3	0.615	0.052	5.65	0.0102

P1: Beira Rio (urban area), P2: Bananal (rural area), P3: Embiral (rural area), P4: Cidelândia (rural area).

Table S14. Results of essential and potentially toxic elements in the waters of the middle Tocantins River throughout 2023.

Elements (mg/L) - February. 2023														
Sampling area		**Al**	**Au**	**Ca**	**Cu**	**Fe**	**In**	**K**	**Mg**	**Na**	**S**	**Se**	**Si**	**Sn**
	BR1	4.9	0.68	8.88	0.276	1.76	3.29	2.04	1.03	0.824	0	1.53	7.71	2.03
P1	BR2	4.00	0.432	7.77	0.149	1.41	2.11	1.62	1.034	0.876	0	1.53	10	1.11
	BR3	4.8	0.166	4	0.147	1.507	3	1.38	1.056	0.805	0	1.3	11	1
	B1	5.36	0.684	44.2	0.306	7.14	2.95	3.95	6.05	2.89	0.235	1.97	32.2	2.14
P2	B2	4.76	0.699	38.1	0.321	6.14	3.18	3.07	5.4	2.5	0.213	2.01	28.8	2.25
	B3	4.66	0.776	24.5	0.348	5.99	3.59	2.81	3.62	1.84	0.0394	2.17	21.8	2.46
	E1	4.67	0.596	66.2	0.248	7.26	2.3	3.53	8.64	4.76	0.393	1.62	37.2	1.83
P3	E2	4.38	0.646	65.1	0.272	7.02	2.55	3.46	8.52	4.51	0.41	1.88	36.7	1.98
	E3	3.83	0.613	66.9	0.264	6.17	2.41	3.43	8.67	4.57	0.434	1.73	36.5	1.9
	V1	4.48	0.578	73	0.213	7.92	2.15	10.2	8.75	6.14	0.442	1.6	37.2	1.72
P4	V2	3.43	0.561	68.3	0.214	6.64	2.06	3.47	8.79	6	0.46	1.49	37.2	1.65
	V3	3.49	0.598	59	0.236	6.78	2.3	3.49	7.86	5.42	0.384	1.67	34.2	1.78

P1: Beira Rio (urban area), P2: Bananal (rural area), P3: Embiral (rural area), P4: Cidelândia (rural area).

Table S15. Results of essential and potentially toxic elements in the waters of the middle Tocantins River throughout 2023.

Elements (mg/L) - March. 2023			
Sampling area		**K**	**Na**
	BR1	0.76	0
P1	BR2	0.74	0
	BR3	0.741	0
	B1	0.837	0.0961
P2	B2	0.825	0.0872
	B3	0.807	0.051
	E1	1.03	0.512
P3	E2	0.959	0.302
	E3	0.87	0.193
P4	V1	1.15	0.486
	V2	1.08	0.466

V3	0.985	0.228

P1: Beira Rio (urban area), P2: Bananal (rural area), P3: Embiral (rural area), P4: Cidelândia (rural area).

Table S16. Results of essential and potentially toxic elements in the waters of the middle Tocantins River throughout 2023.

Elements (mg/L) - April. 2023															
Sampling area		**Al**	**Au**	B	**Ca**	**Cu**	**Fe**	**In**	**K**	**Mg**	**Na**	**S**	**Se**	**Si**	**Sn**
P1	BR1	0.311	0.072	0.0019	7.490	0.040	0.447	0.324	0.345	0.912	0.748	0.119	0.205	3.440	0.234
	BR2	0.336	0.070	0.0002	7.210	0.039	0.507	0.306	0.339	0.898	0.671	0.109	0.190	3.480	0.224
	BR3	0.333	0.074	0.0056	7.350	0.040	0.493	0.320	0.358	0.874	0.786	0.132	0.213	3.400	0.235
P2	B1	0.342	0.069	0	6.700	0.038	0.484	0.314	0.294	0.918	0.354	0.052	0.192	3.610	0.224
	B2	0.341	0.069	0	6.330	0.038	0.487	0.316	0.290	0.880	0.345	0.049	0.194	3.490	0.225
	B3	0.347	0.069	0	6.810	0.038	0.511	0.303	0.296	0.936	0.363	0.051	0.193	3.680	0.221
P3	E1	0.334	0.067	0	7.660	0.038	0.508	0.288	0.308	0.897	0.382	0.076	0.186	3.620	0.211
	E2	0.332	0.055	0	7.540	0.032	0.543	0.225	0.318	0.998	0.406	0.067	0.140	4.000	0.171
	E3	0.336	0.067	0	7.350	0.037	0.499	0.290	0.304	0.818	0.346	0.058	0.178	3.470	0.210
P4	V1	0.345	0.052	0	7.080	0.032	0.653	0.220	0.334	1.000	0.557	0.101	0.133	4.160	0.163
	V2	0.344	0.051	0	7.730	0.031	0.660	0.213	0.333	0.987	0.550	0.085	0.130	4.150	0.158
	V3	0.343	0.056	0	6.130	0.033	0.648	0.232	0.327	0.963	0.529	0.078	0.141	4.050	0.172

P1: Beira Rio (urban area), P2: Bananal (rural area), P3: Embiral (rural area), P4: Cidelândia (rural area).

Table S17. Results of essential and potentially toxic elements in the waters of the middle Tocantins River throughout 2023.

Elements (mg/L) - May. 2023														
Sampling area		**Al**	**Au**	**Ca**	**Cu**	**Fe**	**In**	**K**	**Mg**	**Na**	**S**	**Se**	**Si**	**Sn**
P1	BR1	0.217	0.022	8.820	0.010	0.392	0.058	0.346	1.120	0.656	0.045	0.010	4.450	0.051
	BR2	0.220	0.021	8.380	0.010	0.387	0.056	0.348	1.080	0.625	0.043	0.008	4.360	0.050
	BR3	0.208	0.021	8.880	0.010	0.389	0.052	0.346	1.120	0.658	0.044	0.001	4.450	0.047
P2	B1	0.246	0.020	8.440	0.009	0.423	0.060	0.334	1.100	0.495	0.032	0.009	4.460	0.049
	B2	0.231	0.019	9.230	0.008	0.403	0.049	0.332	1.140	0.522	0.031	0.000	4.500	0.044
	B3	0.228	0.020	8.490	0.009	0.408	0.053	0.332	1.100	0.499	0.029	0.006	4.460	0.045
P3	E1	0.221	0.020	7.750	0.009	0.405	0.048	0.336	1.030	0.492	0.026	0.003	4.660	0.043
	E2	0.232	0.021	7.860	0.009	0.399	0.061	0.334	1.040	0.498	0.026	0.011	4.330	0.049
	E3	0.258	0.021	8.050	0.010	0.423	0.062	0.336	1.050	0.511	0.030	0.002	4.380	0.050
P4	V1	0.222	0.021	7.640	0.010	0.473	0.063	0.351	1.040	0.799	0.059	0.018	4.470	0.051
	V2	0.211	0.022	7.800	0.010	0.468	0.061	0.347	1.040	0.813	0.060	0.009	4.320	0.050
	V3	0.205	0.022	7.690	0.010	0.454	0.058	0.344	1.040	0.801	0.057	0.010	4.330	0.048

P1: Beira Rio (urban area), P2: Bananal (rural area), P3: Embiral (rural area), P4: Cidelândia (rural area).

Table S18. Results of essential and potentially toxic elements in the waters of the middle Tocantins River throughout 2023.

Elements (mg/L) - June. 2023													
Sampling area		**Al**	**Au**	**Ca**	**Cu**	**Fe**	**In**	**K**	**Mg**	**Na**	**Se**	**Si**	**Sn**
P1	BR1	0.0319	0.0265	0.146	0.0192	0	0.132	0.157	0.0515	0.0491	0.0221	0.0028	0.0626

	BR2	0.0935	0.0512	0.639	0.0216	0	0.262	0.249	0.231	0.208	0.0415	0.452	0.164
	BR3	0.0612	0.0394	0.191	0.0143	0	0.198	0.191	0.132	0.11	0.0774	0.148	0.111
	B1	0.0823	0.0426	0.14	0.0156	0.0075	0.212	0.198	0.141	0.0866	0.062	0.211	0.123
P2	B2	0.0264	0.0234	0	0.0058	0	0.116	0.155	0.0361	0.0501	0.0149	0.111	0.0492
	B3	0.0244	0.0225	0	0.0068	0	0.116	0.15	0.0208	0.0386	0.0149	0.001	0.0477
	E1	0.124	0.0467	0.881	0.0174	0.371	0.208	0.212	0.0043	0.0801	0.064	0.0695	0.123
P3	E2	0.0482	0.0314	0.466	0.0067	0.0099	0.137	0.169	0.108	0.0807	0.0437	0.692	0.0732
	E3	0.123	0.0585	0	0.0211	0.0363	0.275	0.203	0.148	0.0666	0.128	0.876	0.171
	V1	0.0517	0.0195	0.0297	0.0051	0.38	0.0982	0.158	0.0213	0.044	0	0	0.0342
P4	V2	0.0158	0.0158	0	0.0022	0.114	0.0776	0.144	0.0213	0.0256	0	0	0.0188
	V3	0	0.0025	0	0.0022	0.0561	0.0776	0.13	0.01	0.0164	0	0	0.0188

P1: Beira Rio (urban area), P2: Bananal (rural area), P3: Embiral (rural area), P4: Cidelândia (rural area).

Table S19. Results of essential and potentially toxic elements in the waters of the middle Tocantins River throughout 2023.

Elements (mg/L) - July. 2023											
Sampling area		**Al**	**Ca**	**Cu**	**Fe**	**K**	**Mg**	**Mn**	**Na**	**S**	**Si**
	BR1	0.182	7.68	0.0176	0.106	0.265	0.816	0.0043	0.708	0.456	4.43
P1	BR2	0.0565	6.69	0.0531	0.147	0.333	0.659	0.0043	1.07	0.104	4.37
	BR3	0.127	9.16	0.0238	0.494	0.33	0.9	0.0043	1.11	0.136	4.65
	B1	0.033	7.2	0.0173	0.0762	0.249	0.812	0	0.465	0.0249	4.5
P2	B2	0	5.76	0.0563	0.214	0.281	0.601	0	0.629	0.0308	4.48
	B3	0.0954	7.09	0.0233	0.307	0.244	0.822	0	0.49	0.0363	4.68
	E1	0.0485	6.51	0.0481	0.336	0.273	1.03	0	0.994	0.0415	4.95
P3	E2	0.0438	5.36	0.0456	0.139	0.254	0.618	0	0.625	0.0466	4.64
	E3	0.0423	6.79	0.0262	0.247	0.256	0.778	0	0.663	0.0321	4.85
	V1	0.0538	6.93	0.0228	0.261	0.267	0.783	0.0093	0.882	0.0822	4.62
P4	V2	0.0825	7.38	0.0329	0.294	0.291	0.789	0.0055	1	0.121	4.64
	V3	0.0833	5.54	0.0607	0.445	0.279	0.602	0.0041	0.956	0.0905	4.49

P1: Beira Rio (urban area), P2: Bananal (rural area), P3: Embiral (rural area), P4: Cidelândia (rural area).

Table S20. Results of essential and potentially toxic elements in the waters of the middle Tocantins River throughout 2023.

Elements (mg/L) - August. 2023									
Sampling area		**Ca**	**Cu**	**Fe**	**K**	**Mg**	**Na**	**S**	**Si**
	BR1	5.7	0.0567	0	0.299	0.621	0.871	0.13	4.37
P1	BR2	14.9	0.0208	6.47	0.659	1.19	3.77	0.443	4.7
	BR3	6.56	0.0159	0	0.254	0.75	0.657	0.042	4.42
	B1	5.34	0.0431	0	0.236	0.637	0.556	0	4.35
P2	B2	6.86	0.0213	0	0.244	0.789	0.473	0	4.37
	B3	6.87	0.0178	0	0.26	0.805	0.472	0	4.59
	E1	5.89	0.0554	0	0.349	0.669	0.932	0.0188	4.33
P3	E2	8.13	0.0145	0	0.333	0.878	1.29	0.0457	4.35
	E3	7.79	0.0084	0	0.287	0.868	0.618	0.0188	4.49
	V1	5.62	0.0419	0.247	0.325	0.648	1.06	0.0685	4.56
P4	V2	6.65	0.0167	0.0935	0.279	0.764	0.865	0.0404	4.44
	V3	7.34	0.0099	0.139	0.279	0.829	0.886	0.0567	4.45

P1: Beira Rio (urban area), P2: Bananal (rural area), P3: Embiral (rural area), P4: Cidelândia (rural area).

Table S21. Results of essential and potentially toxic elements in the waters of the middle Tocantins River throughout 2023.

Elements (mg/L) - September. 2023									
Sampling area		**Ca**	**Cu**	**Fe**	**K**	**Mg**	**Na**	**S**	**Si**
P1	BR1	6.4	0.0487	0.0127	0.272	0.669	0.932	0.129	4.27
	BR2	8.32	0.0205	0	0.278	0.857	1.03	0.121	4.27
	BR3	7.8	0.0187	0.0137	0.25	0.838	0.791	0.725	4.24
P2	B1	6.07	0.0291	0	0.242	0.706	0.637	0.041	4.24
	B2	7.18	0.0178	0	0.228	0.83	0.569	0.254	4.27
	B3	7.74	0.0136	0	0.219	0.889	0.554	0.537	4.33
P3	E1	1.14	0.0349	0	0.154	0.237	0.167	0.088	4
	E2	1.22	0.0454	0	0.184	0.263	0.141	0.273	4.3
	E3	7.64	0.0216	0	0.287	0.866	0.82	0.585	4.31
P4	V1	6.11	0.0387	0.0499	0.256	0.73	0.913	1.34	4.21
	V2	7.22	0.0179	0.0082	0.247	0.843	0.848	0.458	4.28
	V3	7.83	0.0125	0	0.241	0.903	0.807	0.721	4.29

P1: Beira Rio (urban area), P2: Bananal (rural area), P3: Embiral (rural area), P4: Cidelândia (rural area).

Table S22. Results of essential and potentially toxic elements in the waters of the middle Tocantins River throughout 2023.

Elements (mg/L) - October. 2023													
Sampling area		**Al**	**Au**	**Ca**	**Cu**	**Fe**	**In**	**K**	**Mg**	**Na**	**S**	**Si**	**Sn**
P1	BR1	0.0694	0.0123	9.21	0.087	0.128	0.0572	0.497	1.03	2.5	0.177	4.19	0.0402
	BR2	0.0792	0.0152	11	0.0542	0.0782	0.0632	0.448	1.19	2.29	0.211	4.2	0.0519
	BR3	0.0831	0.0169	10.9	0.0554	0.0762	0.0772	0.439	1.18	2.22	0.211	4.21	0.0606
P2	B1	0.0489	0.0078	9.38	0.0729	0.0414	0.0504	0.539	1.07	2.96	0.159	4.13	0.0339
	B2	0.0512	0.0101	10.6	0.0506	0.0304	0.054	0.492	1.19	2.62	0.162	4.16	0.0413
	B3	0.0616	0.0136	10.7	0.0452	0.0358	0.0702	0.466	1.2	2.37	0.118	4.16	0.0548
P3	E1	0.0475	0.006	10.2	0.0654	0.0889	0.0302	0.689	1.14	3.95	0.194	4.13	0.0233
	E2	0.0348	0.0089	11.5	0.0463	0.361	0.0448	0.596	1.25	3.29	0.248	4.08	0.0588
	E3	0.0598	0.0078	9.66	0.0635	0.0683	0.0518	0.556	1.08	3.08	0.251	4.12	0.0353
P4	V1	0.0532	0.0073	11.9	0.0579	0.081	0.0266	1.26	1.28	4.00	0.464	4.19	0.0259
	V2	0.0334	0.0006	12.8	0.0339	0.0653	0	0.679	1.36	3.95	0.186	4.17	0.0058
	V3	0.0631	0.0079	13	0.0449	0.0967	0.0366	0.839	1.36	5.02	0.332	4.2	0.0362

P1: Beira Rio (urban area), P2: Bananal (rural area), P3: Embiral (rural area), P4: Cidelândia (rural area).

Table S23. Results of essential and potentially toxic elements in the waters of the middle Tocantins River throughout 2023.

Elements (mg/L) - November. 2023													
Sampling area		**Al**	**Au**	**Ca**	**Cu**	**Fe**	**In**	**K**	**Mg**	**Na**	**S**	**Si**	**Sn**
P1	BR1	0.0635	0.0169	8.8	0.0646	0.366	0.0701	0.319	1.05	1.2	0.0741	4.23	0.0488
	BR2	0.0351	0.0174	9.52	0.0469	0.157	0.0663	0.288	1.15	1.08	0.0636	4.22	0.0521

	BR3	0.0363	0.018	9.98	0.0475	0.0787	0.0679	0.299	1.18	1.14	0.0785	4.24	0.0558
	B1	0.0478	0.0177	9.48	0.0396	0.062	0.0622	0.278	1.2	0.992	0.039	4.26	0.0522
P2	B2	0.0276	0.0124	7.86	0.0695	0.0373	0.0321	0.319	0.993	1.32	0.0669	4.29	0.0252
	B3	0.0286	0.0172	8.77	0.0545	0	0.0672	0.281	1.14	1.03	0.0414	4.26	0.051
	E1	0.0553	0.0152	8.98	0.0651	0.0651	0.0615	0.314	1.01	1.2	0.0633	4.22	0.0425
P3	E2	0.0939	0.0167	9.07	0.053	0.0543	0.0624	0.301	1.13	1.07	0.0564	4.33	0.0491
	E3	0.0319	0.016	9.24	0.0409	0.0195	0.0566	0.29	1.16	1.08	0.0496	4.24	0.0471
	V1	0.0667	0.0182	8.11	0.0703	0.202	0.0687	0.331	1.01	1.25	0.152	4.37	0.0495
P4	V2	0.034	0.0173	8.79	0.0498	0.0366	0.0697	0.319	1.12	1.23	0.0947	4.34	0.053
	V3	0.0389	0.0165	12.7	0.0417	0.0969	0.0678	0.642	1.35	1.78	0.0934	4.31	0.0537

P1: Beira Rio (urban area), P2: Bananal (rural area), P3: Embiral (rural area), P4: Cidelândia (rural area).

Table S24. Results of essential and potentially toxic elements in the waters of the middle Tocantins River throughout 2023.

Elements (mg/L) - December. 2023											
Sampling area		**Al**	**Au**	**Ca**	**Cu**	**Fe**	**K**	**Mg**	**Na**	**S**	**Si**
	BR1	0.0028	0.0019	10.4	0.0599	0.47	0.366	1.1	1.27	0.398	3.82
P1	BR2	0.0042	0.0016	10.2	0.0389	0.0871	0.263	1.17	0.751	0.124	3.82
	BR3	0.0122	0.0024	11.2	0.0371	0.0682	0.282	1.26	0.825	0.131	3.8
	B1	0.0059	0.0024	8.14	0.0538	0.157	0.318	1.05	0.7	0.0917	3.88
P2	B2	0.0033	0.0021	9.48	0.0364	0.0165	0.237	1.2	0.708	0.0116	3.79
	B3	0	0.0019	9.53	0.037	0.0254	0.287	1.21	0.603	0.0712	3.79
	E1	0.0063	0.0023	7.52	0.055	0.0162	0.256	1.01	0.671	0.0233	3.86
P3	E2	0	0.0033	8.8	0.0379	0.106	0.251	1.14	0.61	0.0343	3.88
	E3	0.0063	0.0029	9	0.035	0.146	0.297	1.16	0.678	0.171	3.8
	V1	0	0.004	7.52	0.0532	0.561	0.264	1.07	0.719	0.055	4.01
P4	V2	0	0.0025	8.45	0.0404	0.0125	0.246	1.13	0.679	0.0545	3.83
	V3	0.0294	0.0024	8.75	0.0358	0.102	0.246	1.16	0.673	0.0392	3.9

P1: Beira Rio (urban area), P2: Bananal (rural area), P3: Embiral (rural area), P4: Cidelândia (rural area).

Table S25. The human health risk assessment indices for cancer risks from ingestion and absorption of studied metals for children and adults.

Parameters	Sampling Points	Children			Adults		
		EXPing	EXPder	THQ	EXPing	EXPder	THQ
Al	*P1*	0.0209	0.0866	0.0273	0.000419	0.0218	0.000185
	P2	0.0215	0.0889	0.0279	0.000432	0.0224	0.000190
	P3	0.0192	0.0790	0.0246	0.000384	0.0199	0.000168
	P4	0.0176	0.0724	0.0226	0.000353	0.0182	0.000155
Cu	*P1*	0.006	*0.0062*	0.0013	0.00073	0.0018	0.000139
	P2	0.0072	*0.0075*	0.0016	0.00087	0.0021	0.000167
	P3	0.006	*0.0062*	0.0013	0.00073	0.0018	0.000139
	P4	0.006	*0.0062*	0.0013	0.00073	0.0018	0.000139
Fe	*P1*	0.00612	0.0577	0.0182	0.00671	0.0163	0.000153
	P2	0.01254	0.1191	0.0377	0.01385	0.0337	0.000316
	P3	0.01356	0.1279	0.0406	0.01406	0.0362	0.000341
	P4	0.01476	0.1395	0.0442	0.01540	0.0394	0.000372
Mg	*P1*	0.0237	0.1205	0.2636	0.000605	0.0341	0.2930
	P2	0.0322	0.1654	0.3605	0.000823	0.0468	0.4005
	P3	0.0395	0.2008	0.4384	0.000995	0.0570	0.4889
	P4	0.0400	0.2035	0.4434	0.00101	0.0578	0.4953
Se	*P1*	0.020	0.0251	0.0034	0.0080	0.0070	0.0045
	P2	0.019	0.0237	0.0032	0.0077	0.0066	0.0043

	P3	0.017	0.0212	0.0028	0.0068	0.0059	0.0038
	P4	0.013	0.0163	0.0022	0.0052	0.0045	0.0029
HI				1.2423			1.5856

P1: Beira Rio (urban area), P2: Bananal (rural area), P3: Embiral (rural area), P4: Cidelândia (rural area). EXP-ing: exposure dose through ingestion of water (mg/kg/day). EXP-der: exposure dose through dermal absorption (mg/kg/day). THQ: Target Hazard Quotient. HI: Hazardous Index.

Table S26. Pearson correlation coefficients for water quality monitoring data of the middle Tocantins River, Maranhão, Brazil.

	TS	TW	pH	COND	SAL	TDS	LDO	TURB	CHLO	ORP	NH_4^+	NO_2	NO_3^-	Al	Au	In	Ca	Cu	Fe	K	Mg	Na	S	Se	Si	Sn
TS		0.211	0.906	0.755	0.803	0.743	0.116	0.347	0.430	0.579	0.699	0.988	0.563	0.196	0.235	0.119	0.659	0.069	0.978	0.498	0.755	0.192	0.032	0.376	0.736	0.137
TW	0.789		0.572	0.772	0.678	0.773	0.256	0.676	0.945	0.787	0.797	0.550	0.316	0.066	0.668	0.077	0.260	0.464	0.476	0.089	0.389	0.029	0.163	0.048	0.308	0.512
pH	-0.094	-0.428		0.815	0.945	0.839	0.753	0.322	0.594	0.376	0.877	0.893	0.784	0.918	0.802	0.942	0.967	0.763	0.982	0.644	0.855	0.796	0.659	0.691	0.972	0.778
COND	0.244	-0.228	-0.185		0.009	0.000	0.848	0.901	0.286	0.417	0.004	0.029	0.241	0.630	0.188	0.761	0.185	0.668	0.052	0.402	0.120	0.669	0.693	0.486	0.143	0.302
SAL	0.197	-0.322	-0.055	**0.991**		0.007	0.841	0.960	0.238	0.338	0.008	0.020	0.233	0.577	0.225	0.711	0.146	0.667	0.026	0.320	0.107	0.601	0.769	0.408	0.107	0.349
TDS	0.257	-0.227	-0.161	**0.999**	**0.993**		0.868	0.927	0.267	0.395	0.002	0.031	0.253	0.640	0.181	0.772	0.189	0.648	0.052	0.400	0.127	0.676	0.687	0.488	0.146	0.294
LDO	-0.884	-0.744	-0.247	0.152	0.159	0.132		0.128	0.566	0.608	0.923	0.649	0.245	0.096	0.573	0.057	0.390	0.123	0.657	0.394	0.414	0.139	0.238	0.289	0.451	0.443
TURB	-0.653	-0.324	-0.678	0.099	0.040	0.073	0.872		0.383	0.319	**0.988**	0.784	0.398	0.393	0.686	0.337	0.642	0.173	0.852	0.785	0.576	0.484	0.559	0.672	0.690	0.612
CHLO	0.569	-0.055	0.406	0.714	0.762	0.733	-0.434	-0.617		0.034	0.227	0.366	0.782	0.949	0.193	0.807	0.554	0.207	0.326	0.594	0.568	0.989	0.544	0.736	0.493	0.244
ORP	-0.420	0.213	-0.624	-0.583	-0.662	-0.605	0.392	0.681	-0.966		0.353	0.454	0.826	0.969	0.374	0.902	0.564	0.289	0.384	0.508	0.628	0.901	0.741	0.640	0.515	0.433
NH_4^+	0.301	-0.203	-0.123	**0.996**	**0.992**	**0.998**	0.077	0.012	0.773	-0.647		0.043	0.289	0.679	0.158	0.816	0.211	0.594	0.060	0.415	0.152	0.710	0.655	0.510	0.165	0.266
NO_2	0.011	-0.450	-0.107	**0.971**	**0.980**	**0.969**	0.351	0.216	0.634	-0.546	**0.957**		0.121	0.419	0.349	0.539	0.069	0.860	0.008	0.237	0.036	0.452	0.931	0.299	0.044	0.492
NO_3^-	-0.436	-0.684	-0.216	0.759	0.767	0.747	0.755	0.602	0.218	-0.174	0.711	0.879		0.138	0.757	0.202	0.041	0.651	0.133	0.165	0.027	0.185	0.669	0.155	0.054	0.922
Al	-0.804	-0.934	0.082	0.370	0.423	0.360	0.904	0.607	-0.051	-0.031	0.321	0.581	0.862		0.760	0.011	0.167	0.351	0.388	0.114	0.237	0.008	0.239	0.057	0.215	0.593
Au	-0.764	-0.332	0.198	-0.812	-0.775	-0.819	0.427	0.314	-0.807	0.626	-0.842	-0.651	-0.243	0.240		0.627	0.667	0.225	0.387	0.914	0.563	0.729	0.209	0.958	0.593	0.016
In	-0.881	-0.923	0.058	0.239	0.289	0.228	0.943	0.663	-0.193	0.098	0.184	0.461	0.798	0.989	0.373		0.256	0.249	0.509	0.184	0.329	0.020	0.168	0.107	0.313	0.472
Ca	0.340	0.740	-0.033	-0.815	-0.854	-0.811	-0.610	-0.358	-0.446	0.436	-0.789	-0.931	-0.959	-0.833	0.333	-0.744		0.838	0.053	0.070	0.019	0.184	0.699	0.092	0.003	0.838
Cu	-0.930	-0.536	-0.237	-0.332	-0.333	-0.352	0.877	0.827	-0.793	0.711	-0.406	-0.140	0.349	0.649	0.775	0.751	-0.162		0.851	0.767	0.874	0.384	0.176	0.623	0.914	0.163
Fe	0.022	0.524	-0.018	**-0.948**	**-0.974**	**-0.948**	-0.343	-0.148	-0.674	0.616	-0.940	**-0.992**	-0.867	-0.612	0.613	-0.491	0.947	0.149		0.177	0.043	0.406	**0.994**	0.243	0.030	0.536
K	0.502	0.911	-0.356	-0.598	-0.680	-0.600	-0.606	-0.215	-0.406	0.492	-0.585	-0.763	-0.835	-0.886	0.086	-0.816	0.930	-0.233	0.823		0.159	0.090	0.461	0.011	0.088	0.914
Mg	0.245	0.611	0.145	-0.880	-0.893	-0.873	-0.586	-0.424	-0.432	0.372	-0.848	-0.964	-0.973	-0.763	0.437	-0.671	0.981	-0.126	0.957	0.841		0.275	0.835	0.183	0.015	0.724
Na	0.808	**0.971**	-0.204	-0.331	-0.399	-0.324	-0.861	-0.516	0.011	0.099	-0.290	-0.548	-0.815	**-0.992**	-0.271	-0.980	0.816	-0.616	0.594	0.910	0.725		0.204	0.038	0.232	0.563
S	**0.967**	0.837	-0.341	0.307	0.231	0.313	-0.762	-0.441	0.456	-0.259	0.345	0.069	-0.331	-0.761	-0.791	-0.832	0.301	-0.824	-0.006	0.539	0.165	0.796		0.356	0.774	0.115
Se	-0.624	**-0.952**	0.309	0.514	0.592	0.512	0.711	0.328	0.264	-0.360	0.490	0.701	0.845	0.943	0.042	0.893	-0.908	0.377	-0.757	-0.989	-0.817	-0.962	-0.644		0.121	0.785
Si	0.263	0.692	-0.028	-0.857	-0.893	-0.854	-0.549	-0.310	-0.507	0.485	-0.835	-0.956	-0.946	-0.785	0.407	-0.687	**0.997**	-0.086	0.970	0.912	0.985	0.768	0.226	-0.879		0.760
Sn	-0.862	-0.488	0.222	-0.698	-0.651	-0.706	0.557	0.388	-0.756	0.567	-0.734	-0.508	-0.078	0.406	0.984	0.528	0.162	0.837	0.464	-0.086	0.276	-0.437	-0.885	0.215	0.240	

TS: Temperature surface, TW: Temperatura water, TDS: Total Dissolved Solids, CON: Conductivity, SAL: Salinity, TURB: Turbity, CHLO: Chlorophyll, LDO: Luminescent Dissolved Oxygen. ORP: Oxidation Reduction Potential. NH_4^+: Ammonium, NO_2: Nitrite, and NO_3^-: Nitrate.

Table S27. Loadings from Principal Component Analysis (PCA).

	**Loadings**		
Parameters	**PC 1**	**PC 2**	**PC 3**
**T surface**	-0.098731	**-0.30391**	0.03989
**T water**	-0.20394	-0.17141	**0.22939**
**pH**	0.010061	0.004281	**-0.5821**
**Conductivy**	**0.21587**	-0.18446	0.12079
**Salinity**	**0.22658**	-0.17605	0.04606
**TDS**	**0.21475**	-0.18904	0.10693
**LDO**	0.16954	**0.23766**	0.16336
**Turbity**	0.10031	0.19939	**0.40822**
**Chlorophyll**	0.11493	**-0.26557**	-0.23363
**ORP**	-0.11352	0.21768	**0.35965**
**NH4+**	**0.20856**	-0.20244	0.083853
**NO2**	**0.24851**	-0.11967	0.079791
**NO3-**	**0.25941**	0.036191	0.14752
**Al**	**0.22868**	0.17336	-0.025265
**Au**	-0.083145	**0.30473**	-0.11497
**In**	0.20547	**0.21222**	-0.012319
**Ca**	**-0.26982**	0.0086509	-0.0021416
**Cu**	0.050256	**0.31052**	0.14958
**Fe**	**-0.25333**	0.11292	-0.0075229
**K**	**-0.25258**	-0.048788	0.1862
**Mg**	**-0.26391**	0.035309	-0.10488
**Na**	**-0.22424**	-0.17397	0.096629
**S**	-0.087887	**-0.29162**	0.18491
**Se**	**0.24762**	0.095227	-0.15827
**Si**	**-0.26836**	0.035027	-0.0043972
**Sn**	-0.036604	**0.31668**	-0.12516

Table S28. Scores from Principal Component Analysis (PCA).

	**PC 1**	**PC 2**	**PC 3**
**P1**	46.649	-24.633	0.20358
**P2**	10.347	43.446	0.66114
**P3**	-18.245	-0.14397	-24.308
**P4**	-38.751	-17.374	15.661

Table S29. Summary from Principal Component Analysis (PCA).

**PC**	**Eigenvalue**	**% variance**
**1**	13.73	52.79
**2**	9.33	35.88
**3**	2.95	11.33
